# Recent Advances in Structure Separation of Single‐Wall Carbon Nanotubes and Their Application in Optics, Electronics, and Optoelectronics

**DOI:** 10.1002/advs.202200054

**Published:** 2022-03-16

**Authors:** Xiaojun Wei, Shilong Li, Wenke Wang, Xiao Zhang, Weiya Zhou, Sishen Xie, Huaping Liu

**Affiliations:** ^1^ Beijing National Laboratory for Condensed Matter Physics Institute of Physics Chinese Academy of Sciences Beijing 100190 China; ^2^ Center of Materials Science and Optoelectronics Engineering and School of Physical Sciences University of Chinese Academy of Sciences Beijing 100049 China; ^3^ Beijing Key Laboratory for Advanced Functional Materials and Structure Research Beijing 100190 China; ^4^ Songshan Lake Materials Laboratory Dongguan Guangdong 523808 China

**Keywords:** electronics, optics, optoelectronics, single‐wall carbon nanotubes, structure separation

## Abstract

Structural control of single‐wall carbon nanotubes (SWCNTs) with uniform properties is critical not only for their property modulation and functional design but also for applications in electronics, optics, and optoelectronics. To achieve this goal, various separation techniques have been developed in the past 20 years through which separation of high‐purity semiconducting/metallic SWCNTs, single‐chirality species, and even their enantiomers have been achieved. This progress has promoted the property modulation of SWCNTs and the development of SWCNT‐based optoelectronic devices. Here, the recent advances in the structure separation of SWCNTs are reviewed, from metallic/semiconducting SWCNTs, to single‐chirality species, and to enantiomers by several typical separation techniques and the application of the corresponding sorted SWCNTs. Based on the separation procedure, efficiency, and scalability, as well as, the separable SWCNT species, purity, and quantity, the advantages and disadvantages of various separation techniques are compared. Combined with the requirements of SWCNT application, the challenges, prospects, and development direction of structure separation are further discussed.

## Graphical abstract

Recent progress in the separation of metal/semiconductor, chiral structures, and enantiomers of single‐wall carbon nanotubes by different techniques and their corresponding applications in optics, electronics, optoelectronics, and bio‐imaging are summarized. The remained challenges and opportunities for this subject are discussed.

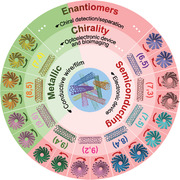

## 1 Introduction

Carbon is an amazing element that has many different stable forms including three‐dimensional diamond, 2D graphene, 1D nanotubes, and 0D fullerene. Research on carbon materials has attracted much attention in the fields of science and industrial technology since the discoveries of fullerene in 1985,^[^ ^1^ ^]^ nanotubes in 1991,^[^ ^2^ ^]^ and graphene in 2004.^[^ ^3^ ^]^ Single‐wall carbon nanotubes (SWCNTs) are some of the most studied carbon materials and were produced for the first time by Iijima, Bethume et al. in 1993.^[^ ^4^, ^5^ ^]^ As a 1D nanomaterial, SWCNTs can be conceptualized as a cylinder rolled up from single‐layer graphene along a certain angle. They usually have a diameter ranging from 0.6 to 2.0 nanometers and a length ranging from several nanometers to centimeters.^[^ ^6^, ^7^ ^]^ For a long time, SWCNTs have provided a material basis for the study of novel physical phenomena, such as the Aharonov Bohm effect,^[^ ^8^, ^9^ ^]^ the quantum Hall effect,^[^ ^10^, ^11^ ^]^ and the Coulomb blockade effect.^[^ ^12^, ^13^ ^]^ In addition, SWCNTs also have broad application prospects in the fields of information, energy, and biomedicine due to their excellent optical, electrical, thermal, and mechanical properties.^[^ ^14^, ^15^, ^16^, ^17^, ^18^, ^19^, ^20^, ^21^, ^22^, ^23^, ^24^, ^25^, ^26^, ^27^, ^28^ ^]^ For example, metallic SWCNTs are ideal conductive materials for fabricating the transparent conductive films used in various flexible electronic devices due to their superior conductivity, flexibility, and stability.^[^ ^18^, ^29^, ^30^, ^31^, ^32^ ^]^ In contrast, semiconducting SWCNTs with nanoscale diameters can be used to fabricate high‐performance, high‐speed and power‐efficient field‐effect transistors (FETs) due to their good size reduction effect in device fabrication, extremely high carrier mobility and structure‐dependent band gaps.^[^ ^14^, ^15^, ^16^, ^19^, ^20^, ^21^, ^22^, ^25^, ^26^, ^27^ ^]^ In addition, semiconducting SWCNTs can also be used for near‐infrared (NIR) biological imaging due to their bright photoluminescence (PL) characteristics within the biological transparency window (700–1400 nm).^[^ ^17^ ^]^

**Figure 1:**
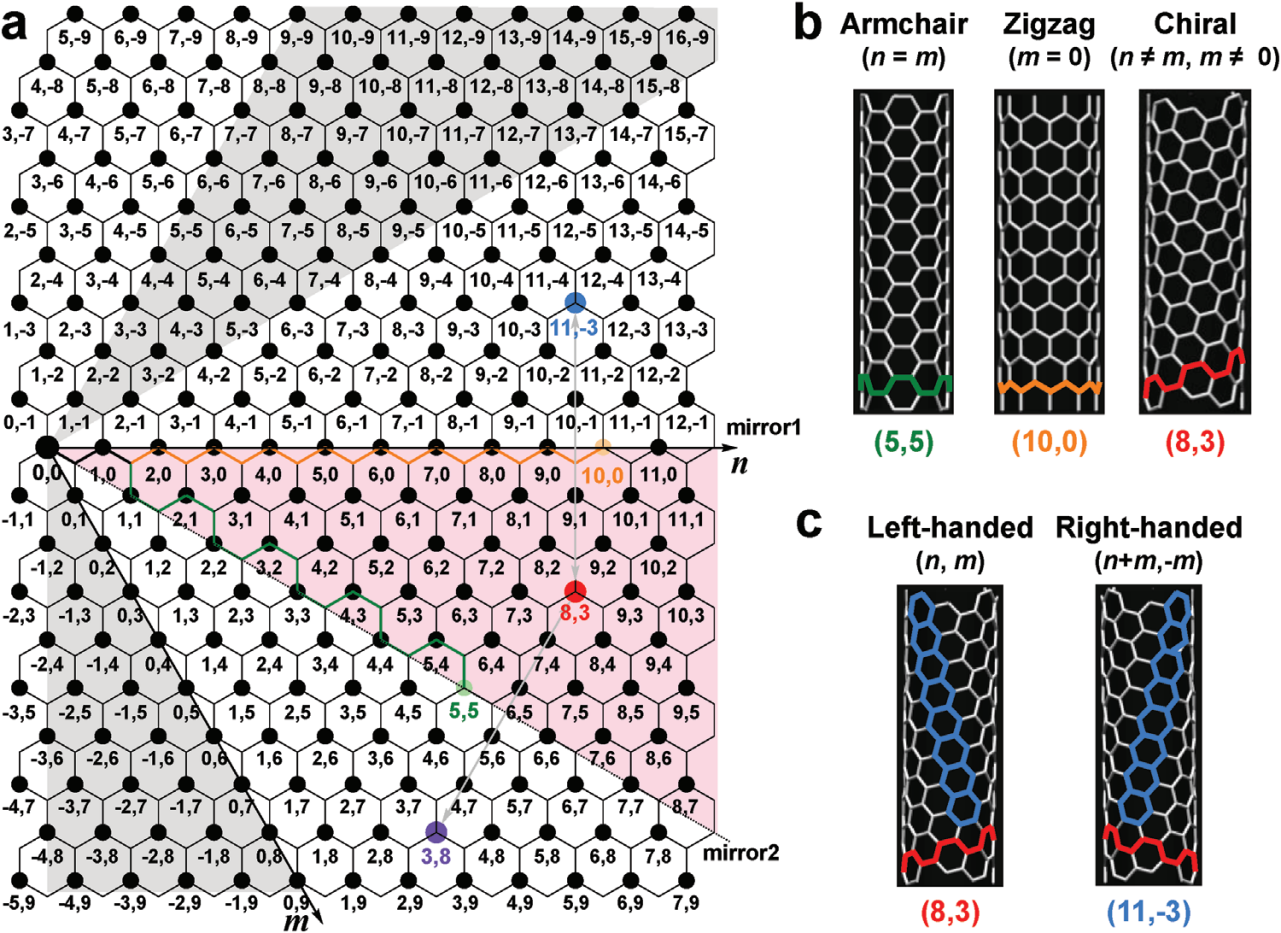
a) Chirality map of SWCNTs, in which (5, 5), (10, 0), and (8, 3) are colored as typical examples of armchair, zigzag, and chiral nanotubes, respectively. A pair of two enantiomers of (8, 3) and (11, −3) (or (3, 8)) are also colored. b) Atomic structures of (5, 5), (10, 0), and (8, 3) nanotubes. c) Atomic structures of (8, 3) and (11, −3) nanotubes, which are the enantiomers of each other.

The unique optoelectronic properties of SWCNTs are derived from their 1D tubular structures with various atomic helical arrangements.^[^ ^6^, ^7^ ^]^ The atomic structure of SWCNTs is widely defined by a pair of integers (*n*, *m*). As shown in **Figure** **1**a, the diameter (*d*_t_) and chiral angle (*θ*) of a nanotube can be calculated by

(1) $$d_{t}=\;0.142\sqrt{3(n^{2}+nm+m^{2}}/\pi$$

and

(2) $$\theta \;=\mathrm{ta}n^{-1}\;\frac{\sqrt{3}m}{2n+m}$$

respectively. The value of *θ* varies within the range of 0–30°. As shown in Figure 1b, three types of SWCNTs can be distinguished depending on their chiral angles: a) Armchair nanotubes (*n = m*), which have a chiral angle of 30°; b) zigzag nanotubes (*m* = 0), which have a chiral angle of 0°; and c) chiral nanotubes (*n* ≠ *m* ≠ 0), which have chiral angles ranging from 0° to 30°. Because the atomic arrangements of the armchair and zigzag nanotubes are symmetrical with respect to the nanotube axis direction, these materials are collectively referred to as achiral nanotubes. Different from achiral species, each specific chiral nanotube has a symmetric mirror structure (different handedness) defined as (*m*, *n*) as an enantiomer (Figure 1c). In the theoretical calculation of a SWCNT, the atomic arrangement of the corresponding enantiomer is sometimes defined as (*n* + *m*, −*m*) instead of (*m*, *n*), in which the indices of two enantiomers are defined to satisfy the condition of 0 ≤ *m* ≤ *n*.^[^ ^33^, ^34^, ^35^ ^]^ However, the two definitions are completely equivalent.

**Figure 2:**
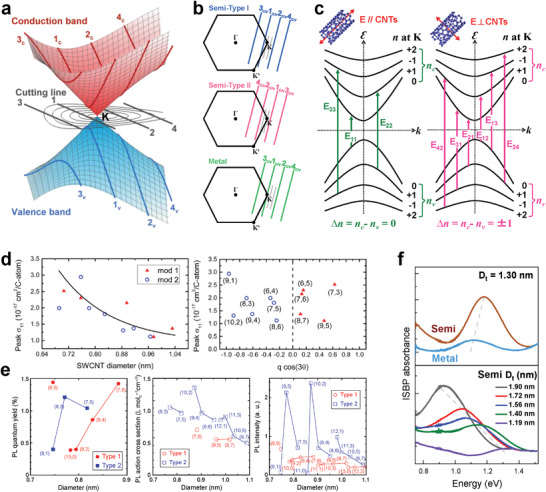
a) Schematic diagram of the conduction and valence band of single‐layer graphene around the K point and cutting lines in the reciprocal space of SWCNTs. b) Reciprocal space of the graphene and cutting lines corresponding to semiconducting Type I and Type II SWCNTs and metallic SWCNTs. c) Energy bands and allowed optical transitions of a semiconducting SWCNT. b,c) Reproduced with permission.^[^ ^40^ ^]^ Copyright 2016, Nature Publishing Group. d) Plots of the S_11_ absorption cross sections of various (*n*,*m*) SWCNTs as a function of SWCNT diameter (left) and qcos(3*θ*) (right). d) Reproduced with permission.^[^ ^49^ ^]^ Copyright 2016, American Chemical Society. e) Plots of the PL quantum yields (left), PL action cross section (middle), and PL intensity (right) of various (*n*,*m*) SWCNTs as a function of SWCNT diameter. e) Reproduced with permission.^[^ ^48^ ^]^ Copyright 2020, American Chemical Society. f) Electronic type and diameter dependence of the ISBPs of SWCNTs. f) Reproduced with permission.^[^ ^50^ ^]^ Copyright 2021, Wiley‐VCH.

The properties of SWCNTs are determined by their structures. Minute differences in the atomic arrangements of SWCNTs cause a dramatic difference in the bandgap structure and thus their electrical and optical properties. For example, where *k* is an integer, SWCNTs with *n* − *m* = 3*k* exhibit metallic conductive behavior, while SWCNTs with *n* − *m* = 3*k* + 1 or 2 exhibit semiconducting conductive behavior.^[^ ^34^ ^]^ Therefore, approximately one‐third of early‐grown SWCNTs without structural control SWCNTs are metallic, and two‐thirds are semiconducting. Furthermore, semiconducting SWCNTs can be divided into Type I and Type II according to mod(2*n* + *m*, 3) = 1 or 2. The metallic and semiconducting SWCNTs have different electronic structures that originate from the hexagonal Brillouin zone of single‐layer graphene (**Figure** **2**a).^[^ ^36^ ^]^ Figure 2b shows the reciprocal space of graphene and a set of cutting lines of semiconducting Type I and Type II SWCNTs and metallic SWCNTs. For a metallic SWCNT, the cutting line corresponding to the first valence and conduction bands crosses the K point. For a semiconducting SWCNT, the cutting line corresponding to the first valence and conduction bands does not cross the K point, which is located on the left side for Type I and the right side for Type II.^[^ ^36^ ^]^ SWCNTs with different atomic arrangements have different electronic structures that directly determine their optical, electrical, and optoelectronic properties. A classic example is that SWCNTs exhibit chirality‐dependent optical transition energies, which have been well summarized by theoretical calculations and experimental observations.^[^ ^37^, ^38^, ^39^, ^40^ ^]^ It is important to note that the allowed optical transitions from the valence band to the conduction band in semiconducting SWCNTs depend on the electric field polarization of the incident light (Figure 2c). When the electric field polarization is parallel to the SWCNT axis, E_ii_ are allowed; when the electric field polarization is perpendicular to the SWCNT axis, E_ij_ transitions are allowed. More details are described in previous reports.^[^ ^40^, ^41^, ^42^ ^]^ In addition, SWCNTs with different chirality species exhibit completely different absorption cross sections (Figure 2d) and PL quantum yields (Figure 2e), even though they have similar diameters.^[^ ^43^, ^44^, ^45^, ^46^, ^47^, ^48^, ^49^ ^]^ Unlike the interband transition E_ii_ or E_ij_, which are from the valence to the conduction bands, intersubband plasmon (ISBP) excitation occurs within the conduction band or the valence band and appears as a new absorption band at NIR wavelengths when the interband transitions are suppressed by electrical gate doping with ionic liquids. Recent studies have demonstrated that the ISBP excitation of SWCNTs is strongly dependent on their electronic structures and diameters (Figure 2f).^[^ ^50^ ^]^

Due to the diversity of structures and properties, high‐end applications of SWCNTs, such as, high‐performance optoelectronic integrated circuits (ICs) and biological imaging, inevitably require species with controllable structures and uniform properties. In the field of semiconductor devices, SWCNTs with specific structures exhibit uniform gate control features due to their uniform band gap.^[^ ^51^ ^]^ A classic example is that Peng et al. recently employed high‐purity semiconducting SWCNTs to fabricate transistors with higher performance than those made of traditional silicon and other semiconductors.^[^ ^52^, ^53^, ^54^ ^]^ In the field of biological imaging, SWCNTs with specific structures show bright PL at specific wavelengths and thus provide high imaging resolution under reduced doses.^[^ ^28^ ^]^ For example, Yomogida et al. demonstrated that single‐chirality (9, 4) SWCNTs with an ≈100‐fold lower injected dose exhibited deeper and brighter NIR biological vascular imaging than pristine HiPco‐SWCNTs.^[^ ^55^ ^]^ More examples are described in later sections. These results have fully demonstrated that the structural control of SWCNTs at the atomic level is critical for the discovery and modulation of the properties of SWCNTs and their applications in different fields. Therefore, over the past two decades, the structural control of SWCNTs has been at the forefront of international research and common topic in the field of SWCNTs.

**Figure 3:**
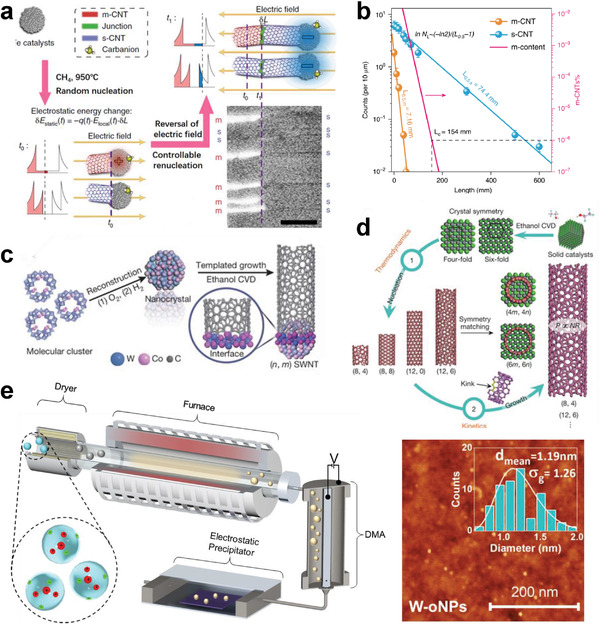
a) Schematic description of the selective synthesis of semiconducting SWCNTs by using electro‐renucleation, and SEM images of the synthesized SWCNTs with chirality ranging from metallic to semiconducting species. a) Reproduced with permission.^[^ ^65^ ^]^ Copyright 2018, Macmillan Publishers Limited, part of Springer Nature. b) Quantity statistics of metallic and semiconducting ultralong nanotubes predicted that the semiconducting percentage reached 99.9999% at a length of 154 mm. Reproduced under the terms of a Creative Commons Attribution 4.0 International License.^[^ ^66^ ^]^ Copyright 2019, The Authors. Published by Springer Nature. c) Schematic of the templated synthesis of SWCNTs with specified chirality using the prepared W—Co nanocrystal catalyst. c) Reproduced with permission.^[^ ^78^ ^]^ Copyright 2014, Springer Nature. d) Schematic description of the two‐step control of SWCNT chirality in the ethanol CVD method. d) Reproduced with permission.^[^ ^80^ ^]^ Copyright 2017, Springer Nature. e) Experimental setup (left) for continuous nanoparticle catalyst generation, size selection, and collection using modified aerosol techniques to achieve near monodispersed refractory W‐based catalysts. Atomic force microscopy (AFM) image (right) of tungsten oxide nanoparticles homogenously deposited on SiO_2_/Si substrates. e) Reproduced with permission.^[^ ^93^ ^]^ Copyright 2020, American Association for the Advancement of Science.

To achieve structural control of SWCNTs, there are two main pathways: one is to directly synthesize SWCNTs with specific structures by controlling growth, and the other is to separate the as‐grown SWCNTs by structure. In the past three decades, although chemical vapor deposition (CVD)^[^ ^56^ ^]^ has been developed as the most mature synthesis technique for producing SWCNTs with high nanotube purity (more than 90%) on an industrial scale,^[^ ^14^ ^]^ the selective growth of SWCNTs in conductivity and diameter remains a major challenge on a large scale due to the complex growth mechanism. To synthesize highly pure semiconducting SWCNTs, scientists have developed various modulation techniques to selectively grow them.^[^ ^57^, ^58^, ^59^, ^60^, ^61^, ^62^, ^63^, ^64^, ^65^ ^]^ For example, Ding et al. synthesized well‐aligned semiconducting SWCNTs in a proportion of 95% via CVD by introducing OH^−^ radicals to selectively etch metallic SWCNTs during growth.^[^ ^59^ ^]^ In 2018, Jiang et al. synthesized nearly defect‐free semiconducting SWCNT arrays with purities up to 99.9% by applying an external electric field to amplify the difference in the renucleation barrier between metallic and semiconducting SWCNTs (**Figure** **3**a).^[^ ^65^ ^]^ In 2019, Wei et al. demonstrated that the atomic assembly rate of semiconducting SWCNTs is 10 times higher than that of metallic SWCNTs; therefore, according to the Schulz‐Flory distribution, when the length of ultralong SWCNTs was greater than 154 mm, the predicted 99.9999% abundance of semiconducting SWCNTs was achieved (Figure 3b).^[^ ^66^ ^]^

As early as 2003, researchers recognized the importance of catalysts on the direct chirality control of SWCNTs during synthesis. Bachilo et al. employed CVD to realize the chirality‐selective synthesis of (6, 5)/(7, 5) SWCNTs using silica‐supported bimetallic CoMo as a catalyst.^[^ ^67^ ^]^ Based on this technique, a series of SWCNT materials named by CoMoCAT, were produced on an industrial scale. In addition, other bimetallic catalysts, such as CoMn, CoCr, FeRu, FeCo, FeCr, FeMn, FeCu, and CoCu, were also employed for the selective growth of various chiral species, promoting the understanding of the roles of each component in bimetallic catalysts.^[^ ^68^, ^69^, ^70^, ^71^, ^72^, ^73^, ^74^, ^75^, ^76^, ^77^ ^]^ In 2014, the chirality‐selective growth of SWCNTs achieved a great breakthrough. Yang et al. synthesized single‐chirality (12, 6) SWCNTs using W‐based bimetallic alloy nanocrystals as catalysts (Figure 3c), and the chirality purity was estimated to be 92% using micro‐Raman spectroscopy.^[^ ^78^ ^]^ By optimizing the growth conditions, they further verified W‐based bimetallic alloy nanocrystals to be valid for the specific growth of (16, 0) and (14, 4) SWCNTs with estimated abundances of 79% and 97%, respectively.^[^ ^79^ ^]^ In 2017, Zhang et al. achieved the direct growth of horizontally aligned arrays of (12, 6) and (8, 4) SWCNTs with purities of 90% and 80%, respectively, by controlling the crystal symmetry matching between the SWCNTs and the surfaces of solid carbide catalysts (Figure 3d).^[^ ^80^ ^]^ In addition to bimetallic catalysts, different kinds of alloys and metal oxides have also been proven to be effective for the chirality‐selective growth of SWCNTs.^[^ ^81^, ^82^, ^83^, ^84^, ^85^ ^]^ In addition, various nanocarbon segments have also been demonstrated to be effective in the epitaxial growth of SWCNTs with specific chirality.^[^ ^86^, ^87^, ^88^, ^89^, ^90^ ^]^ However, the continuous production of chirality‐controlled SWCNTs is a long‐sought goal. Using floating CVD catalysts, Kauppinen et al. realized the direct synthesis of colorful SWCNT thin films by the selective nucleation of SWCNTs.^[^ ^91^ ^]^ Most recently, Zhang et al. achieved the continuous production and sieving of refractory W‐, Mo‐, and Re‐based nanoparticles^[^ ^92^ ^]^ and bimetallic alloy catalysts^[^ ^93^ ^]^ with tiny and near‐monodisperse sizes, which demonstrated the significant effectiveness for obtaining a narrow chirality distribution of SWCNTs (Figure 3e). Although the structure‐controlled growth of SWCNTs has made significant progress, the purity and yield of the grown SWCNTs still do not satisfy the requirements for practical applications in devices.

Compared with structure‐controlled synthesis, post‐synthesis structure separation is another effective method of controlling the structure of SWCNTs, and it is considered to be easier for the large‐scale production of SWCNTs with specific structures. Based on the conductivity and atomic structure of SWCNTs as described above, structure separation can be divided into three stages: metallic/semiconducting separation, chirality separation, and enantiomer separation. With the gradual deepening of the separation stage, higher‐resolution separation techniques are required. The metallic/semiconducting separation of SWCNTs started in 2003. Krupke et al. developed a dielectrophoresis method of separating metallic SWCNTs.^[^ ^94^ ^]^ When a mixed SWCNT solution was dropped onto the microelectrode arrays with an external applied electric field, the metallic SWCNTs with larger dipole moments moved faster and then deposited on the electrodes because of the vast difference in the dielectric constants of the metallic and semiconducting SWCNTs. Although dielectrophoresis has demonstrated high selection for metallic SWCNTs, microelectrode fabrication and the use of an external electric field limit the separation scale. In the same year, Strano et al. selectively reacted diazonium reagents with metallic SWCNTs and enriched the semiconducting SWCNTs.^[^ ^95^ ^]^ Subsequently, Hassanien et al. used hydrogen plasma to selectively etch metallic SWCNTs,^[^ ^96^ ^]^ and Zhang et al. used SO_3_ to selectively etch semiconducting SWCNTs,^[^ ^97^ ^]^ enriching the semiconducting or metallic SWCNTs. However, both covalent reactions and etching can dramatically destroy the natural atomic arrangement of the obtained SWCNTs, which inevitably damages their inherent physical and mechanical performance. Moreover, these techniques did not exhibit chirality or enantiomer selectivity, so they are not widely used for the structure separation of SWCNTs.

In contrast to the above mentioned methods, liquid separation methods such as polymer wrapping,^[^ ^98^ ^]^ density gradient centrifugation (DGU),^[^ ^99^, ^100^ ^]^ ion‐exchange chromatography (IEX),^[^ ^101^ ^]^ gel chromatography,^[^ ^102^ ^]^ and aqueous two‐phase (ATP)^[^ ^103^ ^]^ methods exhibit high structural resolution for SWCNTs, which can not only separate metallic/semiconducting SWCNTs but also separate single‐chirality species and even their enantiomers through the precise modulation of interactions of polymers, DNA, or surfactants with SWCNTs. The interaction between these molecules and SWCNTs is a physical adsorption process that does not damage the structure of the SWCNTs. More importantly, these separation techniques have been proven to be efficient and reliable pathways for producing SWCNTs with specific structures on a large scale. Attributed to the rapid development of various separation techniques, great progress has been made in the study of the properties of SWCNTs and their applications in optoelectronics and bioimaging.^[^ ^14^, ^15^, ^16^, ^17^, ^20^, ^21^, ^23^, ^24^, ^25^, ^26^, ^27^, ^28^ ^]^

In this review, we introduce recent progress in the structure separation of SWCNTs using these liquid phase separation techniques and studies of the properties and applications of separated SWCNTs. Although several reviews on the structure separation of SWCNTs have been published,^[^ ^104^, ^105^, ^106^, ^107^, ^108^, ^109^, ^110^, ^111^, ^112^, ^113^, ^114^ ^]^ the most recent progress is still lacking due to the rapid development of separation techniques. Furthermore, systematic reviews of recent advances in studies of the properties and the application of sorted SWCNTs in optoelectronics are rarely reported. Herein, this review has been divided into three sections: 1) Separation of metallic/semiconducting SWCNTs and their application in electronics, 2) separation of single‐chirality SWCNTs and their application in optics, optoelectronics, and bioimaging, and 3) separation of enantiomeric SWCNTs and their application in biomolecular detection. In each section, we introduce the typical separation methods and the characteristics of each of them, including the separation procedure, efficiency, and scalability, as well as, the separable SWCNT species, purity, and quantity. Furthermore, we review the progress in the study of the properties and applications of the corresponding separated SWCNTs in electronics, optics or optoelectronics. Finally, we discuss the current challenges and opportunities for the preparation and application of SWCNTs.

## 2 Strategies for Dispersing Single‐Wall Carbon Nanotubes

The mono‐dispersion of SWCNTs in an aqueous or organic solution is necessary for their structure separation by liquid phase separation methods.^[^ ^115^ ^]^ However, the as‐grown SWCNTs usually exist in the form of bundles due to the strong van der Waals interactions. How to efficiently disperse the as‐grown SWCNT bundles into individuals suspended in aqueous or organic solution has been a hot issue in the fields of SWCNTs. The key to achieve the efficient de‐bundling of as‐grown SWCNTs is to resolve the issues on poor solubility and low dispersibility of SWCNTs in aqueous or organic solutions due to their hydrophobic nature.

In the past decades, covalent and non‐covalent modifications by various dispersants have been reported to improve the hydrophilicity, solubility, and stability of SWCNTs in solution.^[^ ^95^, ^116^, ^117^ ^]^ The covalent modification involves the chemical bonding of different functional groups onto *π*‐conjugated skeleton of SWCNTs, and thus effectively improves the wettability and solubility of SWCNTs, promoting their dispersion in various solvents.^[^ ^118^ ^]^ Although some new features may be created for the covalently functionalized SWCNTs, their intrinsic physical and mechanical properties will be changed and even be degraded due to structural damage originated from chemical reaction. Therefore, the dispersed SWCNTs by the covalent modification have rarely been reported for the subsequent structure separation of SWCNTs.

**Figure 4:**
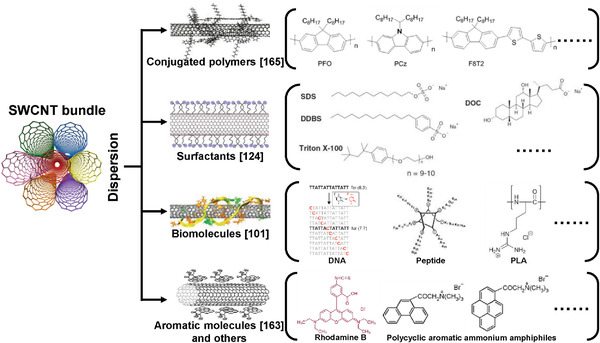
Summary of dispersant molecules used for non‐covalent modification and dispersion of SWCNTs. The images of nanotubes wrapped by conjugated polymers, surfactants, biomolecules, and aromatic molecules are successively reproduced with permission. Reproduced with permission.^[^ ^165^ ^]^ Copyright 2011, The Chemical Society of Japan; Reproduced with permission.^[^ ^124^ ^]^ Copyright 2013, American Chemical Society; Reproduced with permission.^[^ ^101^ ^]^ Copyright 2009, Springer Nature; Reproduced with permission.^[^ ^163^ ^]^ Copyright 2006, Wiley‐VCH.

In contrast, the non‐covalent modification through the physical adsorption of dispersants such as conjugated polymers,^[^ ^98^, ^119^ ^]^ surfactants,^[^ ^120^, ^121^, ^122^, ^123^, ^124^ ^]^ biomolecules,^[^ ^125^, ^126^, ^127^, ^128^, ^129^, ^130^ ^]^ aromatic small molecules,^[^ ^131^, ^132^ ^]^ is considered to be a more moderate and effective approach (a summary of used dispersant molecules is shown in **Figure** **4**), which preserves the intrinsic properties of SWCNTs. The dispersant molecules could be replaced or removed by suitable processes.^[^ ^133^, ^134^ ^]^ More importantly, the mono‐dispersion of SWCNTs prepared by non‐covalent approaches exhibits the good compatibility with various liquid phase separation techniques including polymer wrapping, IEX, DGU, gel chromatography, and ATP, in which the structure separation of SWCNTs such as electrical type, chirality, and enantiomer is achievable. Therefore, in this section we focus on the dispersion of SWCNTs by the non‐covalent modification based on conjugated polymers, surfactants, and biomolecules.

### 2.1 Conjugated Polymers

Conjugated polymers including polyfluorene, polythiophene, and polycarbazone, exhibit a strong ability to disperse SWCNTs in organic solvents.^[^ ^98^, ^111^, ^115^, ^119^ ^]^ The basic principle is that SWCNTs are wrapped by conjugated polymer backbone through *π*–*π* interactions. Compared with other dispersant agents, such as, surfactants and biomolecules described below, a unique advantage of conjugated polymers is that the structural selectivity in electrical type, diameter, chirality, and even enantiomer of SWCNTs could be achieved during the dispersion process, which endows conjugated polymers with the ability to efficiently separate the structures of SWCNTs. For this, various conjugated polymers have been designed and synthesized for the dispersion and separation of SWCNTs.^[^ ^111^, ^115^ ^]^ Previous experimental and theoretical studies revealed that the dispersion and separation of SWCNTs by conjugated polymers strongly depend on their side chains, skeleton structure, molecular weight, and solvent,^[^ ^135^, ^136^, ^137^, ^138^, ^139^, ^140^, ^141^ ^]^ which determine the binding energy between polymers and SWCNTs and the polymer conformation onto nanotube surfaces.^[^ ^115^ ^]^ These findings lay an important foundation for the subsequent structure separation of SWCNTs based on conjugated polymers. More details are described in later sections.

### 2.2 Surfactants

The surfactants have been demonstrated to be important dispersants for the dispersion of SWCNTs in aqueous solution.^[^ ^115^ ^]^ The available surfactants can be classified into ionic and nonionic surfactants, in which ionic surfactants include anionic,^[^ ^120^, ^123^ ^]^ cationic,^[^ ^121^, ^142^ ^]^ and zwitterionic surfactants.^[^ ^143^ ^]^ Although both ionic and nonionic surfactants can disperse SWCNTs in aqueous solution, the interaction mechanism is vastly different. The ionic surfactants generally contain the hydrophilic and hydrophobic parts, in which the hydrophobic part (e.g., alkyl chain) is usually arranged along the nanotube sidewalls and prevents the aggregation, while the hydrophilic part (ionized functional group) toward the outside of nanotube.^[^ ^112^, ^115^ ^]^ The SDS is the earliest reported surfactant for dispersion of SWCNTs in aqueous solution,^[^ ^123^ ^]^ but its dispersion efficiency is limited. Later on, other several typical surfactant molecules, such as, sodium dodecyl benzene sulfonate (SDBS), SC, and DOC have also been proven to be effective for SWCNT dispersion.^[^ ^120^, ^144^, ^145^ ^]^ For the nonionic surfactants, such as Triton and Briji, their dispersion capabilities appear to be dominantly affected by the long or branched disordered polar chains.^[^ ^121^, ^122^ ^]^ The nonionic surfactants with larger molecular weights enable to disperse SWCNTs more efficiently due to the enhanced steric stabilization by longer polymeric groups.^[^ ^122^ ^]^ A quantitative comparison of a wide variety of surfactants for their dispersion efficiency of SWCNTs in water revealed that bile salts were exceptionally effective due to the formation of very regular and stable micelles around the nanotubes.^[^ ^122^ ^]^ Furthermore, the surfactant conformation onto nanotube surfaces is found to be sensitive to the surfactant component and concentration,^[^ ^146^, ^147^ ^]^ pH,^[^ ^148^ ^]^ temperature,^[^ ^149^ ^]^ which strongly affect the structural selectivity in various liquid phase separation methods.

### 2.3 Biomolecules

In addition to conjugated polymers and surfactants, the biomolecules including DNA,^[^ ^125^, ^126^, ^127^ ^]^ nucleotides,^[^ ^128^, ^129^, ^130^ ^]^ polyarginine,^[^ ^150^ ^]^ and proteins,^[^ ^151^, ^152^ ^]^ have also exhibited the strong ability to disperse SWCNTs. Among these biomolecules, DNA is a typical biomolecule that was first used by Zheng et al. in 2003 for the dispersion of SWCNTs.^[^ ^125^ ^]^ They reported that the bundled SWCNTs can be effectively dispersed in aqueous solution of ssDNA under sonication treatment. Based on molecular simulation, they further proposed that DNA binds to individual SWCNTs through *π*‐stacking, resulting in a helical wrapping around the nanotube surfaces. Actually the helical wrapping model of ssDNA around nanotubes was found to be dependent on the specific DNA sequence, nanotube diameter and electrical type, indicating the feasibility for the structure separation of SWCNTs by DNA.^[^ ^126^ ^]^ These pioneering works lead to a series of DNA‐based dispersion and separation of SWCNTs in aqueous and organic solutions.^[^ ^127^, ^153^, ^154^, ^155^, ^156^, ^157^ ^]^ Based on the selective interaction between DNA sequences and SWCNTs, the DNA‐assisted separation techniques including IEX, DGU, and ATP were developed. More details are described in later sections. Similar to DNA, nucleotides have also been reported to wrap individual SWCNTs in aqueous solution through a helical assembly around nanotube surfaces, which imparts the effective dispersion of SWCNTs.^[^ ^128^ ^]^ The formation of a helical ribbon of flavin mononucleotide (FMN) around nanotubes was attributed to the cooperative hydrogen bonding between adjacent flavin moieties under concentric *π*–*π* interactions with nanotube surfaces.^[^ ^128^ ^]^ Although the nucleotides have demonstrated certain ability for the structure recognition of SWCNTs in subsequent studies,^[^ ^129^, ^158^, ^159^, ^160^, ^161^ ^]^ their efficiencies for dispersion and separation are still limited, compared with the widely used ssDNA and conjugated polymers mentioned above.

### 2.4 Other Dispersants

In addition to the dispersants mentioned above, other dispersant molecules such as aromatic molecules (rhodamine B, fluorescein, etc.) and organic solvents (*N*‐methyl‐2‐pyrrolidone, *N*,*N*‐dimethylformamide, etc.) have also been reported to be used for the dispersion of raw SWCNT materials through *π*–*π* or cation–*π* interaction,^[^ ^132^, ^162^, ^163^, ^164^ ^]^ but the lack of apparent structure selectivity of SWCNTs limits their further applications in subsequent structure separation. Despite this, these studies on the dispersion of SWCNTs in aqueous and organic systems based on various dispersants lay an important foundation for property studies and application exploration of isolated SWCNTs.

### 2.5 Dispersion Ways

**Figure 5:**
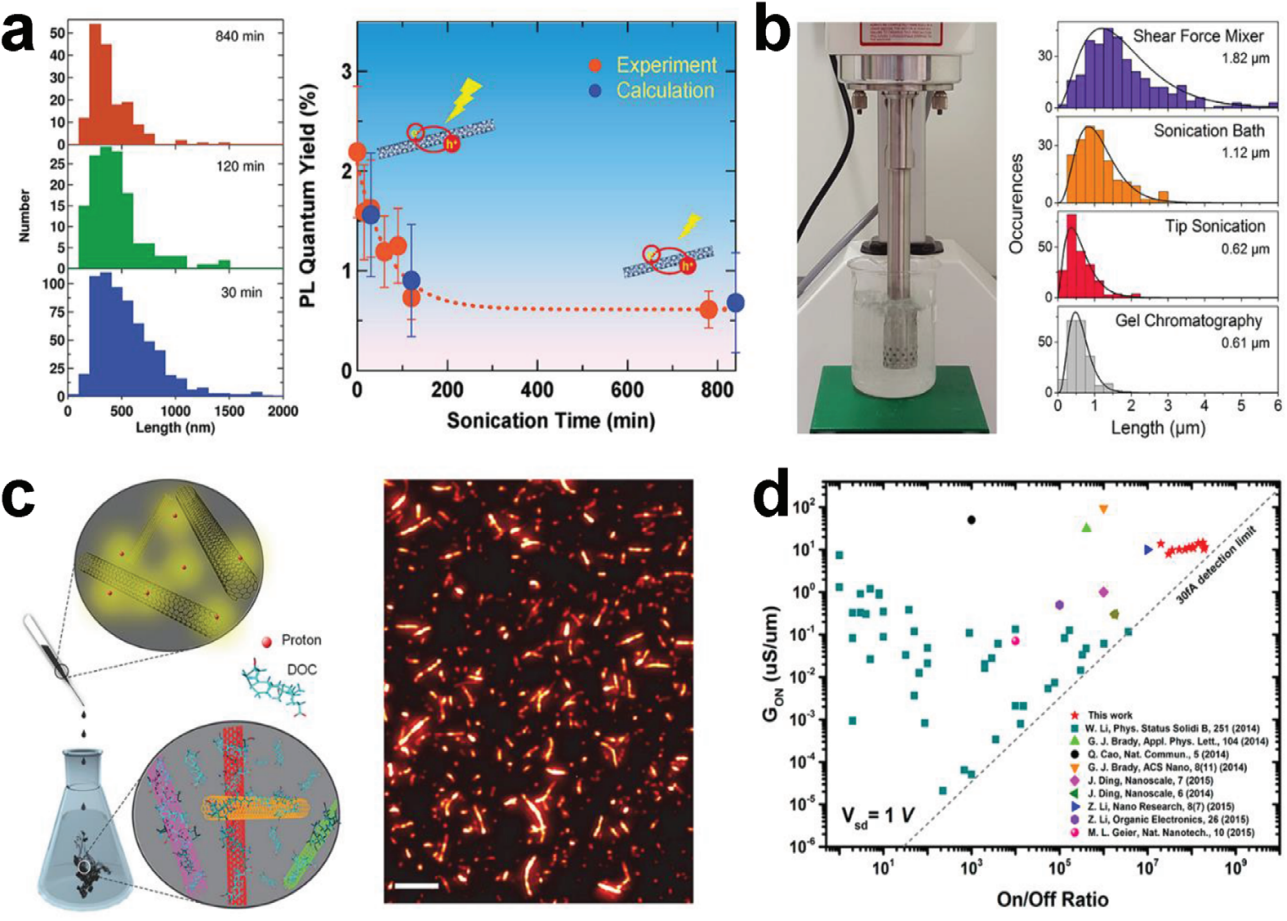
a) Histograms of nanotube length (left) and PL quantum yields of (8, 6) SWCNTs (right) for different sonication times (30, 120, and 840 min). Red and blue dots indicate experimental and calculation results, respectively. a) Reproduced with permission.^[^ ^175^ ^]^ Copyright 2012, American Chemical Society. b) SFM (left) used for dispersion of SWCNTs and length distribution (right) of sorted (6,5) SWCNTs using different dispersion methods. b) Reproduced with permission.^[^ ^178^ ^]^ Copyright 2016, Elsevier Ltd. c) Schematic illustration (left) of the neutralization of a SWCNT‐superacid suspension with DOC/NaOH aqueous solution. PL images (right) of the sorted (6,5)‐SWCNTs using dispersion method of superacid‐surfactant exchange. Scale bar is 10 µm. c) Reproduced with permission.^[^ ^181^ ^]^ Copyright 2017, American Chemical Society. d) Comparison of reported results for on‐state conductance per micron channel width (*G*_ON_) versus On/Off ratio. Red stars indicate the data of all 12 devices prepared in this work. d) Reproduced with permission.^[^ ^168^ ^]^ Copyright 2016, American Chemical Society.

In addition to the dispersant molecules mentioned above, an external force such as, stirring or sonication is also indispensable for the dispersion of SWCNTs. As a typical way, the sonication is widely used to disperse the SWCNT bundles in solution containing the specific dispersant molecules, achieving the wrapping effect of dispersants around nanotube surfaces. Higher dispersion efficiency could be achieved by increasing the ultrasonic power and dispersion time. Unfortunately, the vigorous sonication process inevitably shortens SWCNTs. After dispersion, the length of isolated SWCNTs is usually reduced to the order of hundreds of nanometers, which is much shorter than that of as‐grown SWCNTs (from a few micrometers to tens of micrometers). The shortening effect induced by ultrasonic process can be interpreted as that the local high pressure and friction caused by ultrasonic cavitation damage the carbon lattice, resulting in increasing the defect density on the surface of SWCNTs or even cutting into multi short segments, and thus degrading their intrinsic electrical, optical, and mechanical properties.^[^ ^166^, ^167^, ^168^, ^169^, ^170^, ^171^, ^172^, ^173^, ^174^, ^175^ ^]^ This is one of most important reasons why the films prepared by the solution‐dispersed SWCNTs show higher resistance than those by growth.^[^ ^176^, ^177^ ^]^ Additionally, in SWCNT‐based thin‐film transistors (TFTs), shorter SWCNTs necessitate more intertube charge transfers, which inevitably reduce the on‐state current, on/off ratio, and mobility of the devices.^[^ ^166^ ^]^ As shown in **Figure** **5**a, the PL quantum yield of SWCNTs decreases with increasing sonication time due to an increase in the density of exciton quenching sites.^[^ ^175^ ^]^

To reduce the shortening effect of SWCNTs, high‐efficiency and less‐damage dispersion approaches are highly desirable. Zaumseil et al. developed a simple high‐speed shear force mixing to disperse SWCNTs with poly [(9, 9‐dioctylfluorenyl‐2, 7‐diyl)‐alt‐*co*‐(6,60‐2,20‐bipyridine)] (PFO‐BPy) in toluene.^[^ ^178^ ^]^ The selectively dispersed monochiral (6, 5) SWCNTs show an average length of 1.82 µm and a PL quantum yield of 2.3%, indicating less damage than conventional sonication (Figure 5b).^[^ ^178^ ^]^ Such a dispersion process also demonstrated its effectiveness for the dispersion of layered materials, such as, graphene^[^ ^179^ ^]^ and MoS_2_.^[^ ^180^ ^]^ Most recently, Wang et al. reported that the raw SWCNT materials dissolved in superacid (chlorosulfonic acid) become incredibly easy to disperse in conventional aqueous solution of DOC surfactant containing NaOH for neutralization of the superacid.^[^ ^181^ ^]^ The average length of dispersed SWCNTs is more than 350% longer than that prepared by sonication process (Figure 5c).^[^ ^181^ ^]^ The electrical and optical measurements indicate that such a superacid‐surfactant exchange is nondestructive for SWCNTs. Instead of these ingenious dispersion processes, length sorting based on various separation methods is alternative way to obtain long SWCNTs with excellent electrical and optical properties.^[^ ^167^, ^168^, ^169^, ^182^ ^]^ For example, Kappes et al. employed size‐exclusion chromatography method to obtain the semiconducting SWCNTs with the average length of ≈1 µm. The corresponding TFTs show improved hole mobility of 297 cm^2^ V^−1^ and on/off ratio of 2 × 10^8^ (Figure 5d).^[^ ^168^ ^]^ More efficient and non‐destructive dispersion methods still remains a challenge for improving the properties of SWCNTs.

## 3 Separation of Metallic/Semiconducting Single‐Wall Carbon Nanotubes and Their Applications

**Figure 6:**
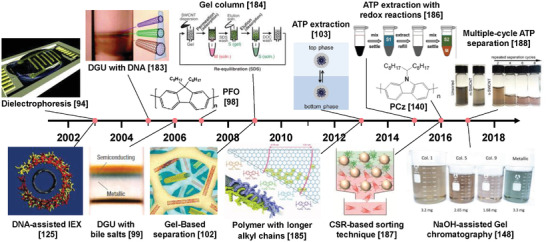
Timeline of separation of metallic/semiconducting SWCNTs. Reproduced with permission from the following: Reproduced with permission.^[^ ^94^ ^]^ Copyright 2003, American Association for the Advancement of Science; Reproduced with permission.^[^ ^125^ ^]^ Copyright 2003, Nature Publishing Group; Reproduced with permission.^[^ ^183^ ^]^ Copyright 2005, American Chemical Society; Reproduced with permission.^[^ ^99^ ^]^ Copyright 2006, Nature Publishing Group; Reproduced with permission.^[^ ^98^ ^]^ Copyright 2007, Nature Publishing Group; Reproduced under the terms of a Creative Commons Attribution 4.0 International License.^[^ ^184^ ^]^ Copyright, 2009, The Japan Society of Applied Physics; Reproduced with permission.^[^ ^102^ ^]^ Copyright 2009, American Chemical Society; Reproduced with permission.^[^ ^103^ ^]^ Copyright 2013, American Chemical Society; Reproduced with permission.^[^ ^185^ ^]^ Copyright 2013, Wiley‐VCH; Reproduced with permission.^[^ ^186^ ^]^ Copyright 2015, American Chemical Society; Reproduced with permission.^[^ ^140^ ^]^ Copyright 2016, Wiley‐VCH; Reproduced with permission.^[^ ^187^ ^]^ Copyright 2016, American Chemical Society; Reproduced with permission.^[^ ^188^ ^]^ Copyright 2017, The Royal Society of Chemistry; Reproduced with permission.^[^ ^148^ ^]^ Copyright 2017, Wiley‐VCH.

The separation of metallic and semiconducting SWCNTs is the foundation for the structure separation of SWCNTs. The semiconducting purity of SWCNTs is the most important factor affecting their application in electronics. Therefore, the separation of ultrapure semiconducting SWCNTs has been a long‐term pursuit in the separation of metallic and semiconducting SWCNTs. To separate high‐purity semiconducting SWCNTs, various separation techniques have been developed in recent years, mainly including polymer wrapping, IEX, DGU, gel chromatography, and ATP (as shown in **Figure** **6**). The common feature of these separation techniques is the selective coating of metallic and semiconducting SWCNTs with polymers, surfactants, or DNA sequences. Each separation method also has its own unique separation characteristics and separation mechanisms that determine the separation efficiency, purity and yield of metallic or semiconducting SWCNTs. The details are described in the following sections.

### 3.1 Polymer Wrapping‐Based Metallic/Semiconducting Separation

Polymer wrapping is a simple, rapid, and scalable pathway to achieve metallic/semiconducting separation of SWCNTs, which mainly involves two steps: Dispersion and concentration. In this method, polymers are used to selectively wrap and disperse the semiconducting SWCNTs, while the undispersed metallic SWCNTs are removed following the concentration process. Since 2001, several polymers, such as polyvinyl pyrrolidone, octadecylamine, and porphyrins, have been found to selectively disperse semiconducting SWCNTs.^[^ ^135^, ^136^, ^137^, ^138^ ^]^ However, the selectivity for semiconducting SWCNTs is not high enough, leading to the low purity of separated semiconducting SWCNTs.

**Figure 7:**
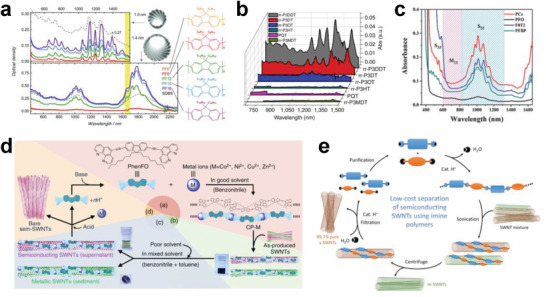
a) Optical absorption spectra of small‐diameter HiPco (average diameter of ≈1 nm) and large‐diameter arc plasma jet (average diameter of ≈1.4 nm) SWCNTs dispersed with different derivatives of polyfluorene in toluene (color curves) and SDBS in water (dashed curves) for comparison. a) Reproduced with permission.^[^ ^185^ ^]^ Copyright 2013, Wiley‐VCH. b) Optical absorption spectra of various polythiophene–SWCNT suspensions after centrifugation. b) Reproduced with permission.^[^ ^191^ ^]^ Copyright 2011, Macmillan Publishers Limited. c) Optical absorption spectra of separated semiconducting SWCNTs dispersed with PCz, PFO, PFBP, and F8T2. c) Reproduced with permission.^[^ ^140^ ^]^ Copyright 2016, Wiley‐VCH. d) A method for the separation of semiconducting SWCNTs by using removable solubilizers. d) Reproduced with permission.^[^ ^192^ ^]^ Copyright 2014, Macmillan Publishers Limited. e) Schematic of the separation cycle of semiconducting SWCNTs. e) Reproduced with permission.^[^ ^193^ ^]^ Copyright 2016, American Chemical Society.

In 2007, Nicholas et al. found that the polymer poly(9,9‐dioctylfluorenyl‐2,7‐diyl) (PFO) exhibited higher selectivity for semiconducting SWCNTs.^[^ ^98^ ^]^ Subsequently, a large number of PFO derivatives, fluorene‐ and carbazole‐based polymers, and their copolymers were developed to improve the selective dispersion of semiconducting SWCNTs.^[^ ^107^, ^111^, ^189^, ^190^ ^]^ For example, based on the separation of small‐diameter (0.8–1.2 nm) semiconducting with PF8 (commonly known as PFO), PF12, PF15, and PF18 with longer alkyl chains were further developed for the separation of SWCNTs with diameters larger than 1.2 nm (**Figure** **7**a).^[^ ^185^ ^]^ Bao et al. significantly improved the separation yield of semiconducting SWCNTs (up to 31%) by increasing the side chain length of polythiophene (Figure 7b); this method was effective because the increase in the surface contact area between the nanotube and the side chain of polymers.^[^ ^191^ ^]^ Later, Gu et al. synthesized a highly soluble carbazole‐based homopolymer with a 1‐octylonoyl sidechain attached onto the N atom (PCz) to separate SWCNTs.^[^ ^140^ ^]^ Both spectroscopic characterization and performance measurement of the TFT device demonstrated that the purities of both small‐ and large‐diameter semiconducting SWCNTs (0.8–1.7 nm) were improved to 99.9% (Figure 7c).^[^ ^140^ ^]^ To improve the purity of semiconducting SWCNTs, the Peng's group further developed multiple dispersion and separation processes based on the conjugated polymer PCz for the separation of semiconducting SWCNTs.^[^ ^54^ ^]^ With this technique, the purity of semiconducting SWCNTs was improved to be higher than 99.999%, which is the highest purity of semiconducting SWCNTs reported previously and satisfies the application of SWCNTs in large‐scale ICs.^[^ ^54^ ^]^

Semiconducting SWCNTs separated by polymer wrapping methods are inevitably coated by a layer of polymers, which dramatically increase the resistance between electrodes and SWCNTs and degrade the performance. The effective removal of wrapped polymers from nanotube surfaces is an important issue for the practical applications of separated semiconducting SWCNTs. To address this issue, Toshimitsu et al. synthesized a PFO‐like metal‐coordination polymer for the separation of semiconducting SWCNTs.^[^ ^192^ ^]^ After separation, this polymer can be depolymerized into monomers through the addition of a protic acid (Figure 7d).^[^ ^192^ ^]^ Lei et al. synthesized another removable conjugated polymer with imine linkages, through which the semiconducting purity of the separated SWCNTs was higher than 99.7%. After separation, the polymers that coat the SWCNTs can be depolymerized into monomers by breaking imine bonds under mildly acidic conditions (Figure 7e).^[^ ^193^ ^]^ Most recently, Xu et al. synthesized several degradable fluorene‐ and carbazole‐based copolymers interlinked by imine bonds for the selective separation of semiconducting SWCNTs.^[^ ^194^ ^]^ Moreover, the released dispersant‐free semiconducting SWCNTs can be easily redispersed by different polymers, which enables the efficient and convenient fabrication of SWCNT devices.^[^ ^194^ ^]^

The high efficiency of the separation of high‐purity semiconducting SWCNTs by polymer wrapping methods and the removal of polymers around the separated SWCNTs provide an important foundation for the preparation of high‐performance carbon‐based electronic devices. In contrast to selective dispersion of semiconducting SWCNTs by polymer wrapping methods, Adronov et al. demonstrated that the PF12 polymer can selectively disperse metallic SWCNTs.^[^ ^195^ ^]^ The selectivity of PF12 toward metallic SWCNTs is dependent on the ratio of metallic/semiconducting SWCNTs present in the raw materials, polymer concentration, and solvent species, which provides a possible strategy for further separating single‐chirality metallic SWCNTs.^[^ ^195^ ^]^

### 3.2 Ion‐Exchange Chromatography‐Based Metallic/Semiconducting Separation

IEX is a chromatographic method in which an ion exchanger is used as a stationary phase, and separation is carried out based on the difference in affinity between the component ions in the mobile phase and the counter ion on the exchanger. IEX has been widely used for the separation of charged molecules, including large proteins, small nucleotides, and amino acids.^[^ ^196^ ^]^ In 2003, Zheng et al. applied the IEX method to the structure separation of DNA‐wrapped SWCNTs.^[^ ^125^ ^]^ This pioneering work opens the door for DNA‐assisted structure separation of SWCNTs. Subsequently, they revealed that different sequences of DNA are highly selective for the different chiral structures of SWCNTs, which results in the structure separation of SWCNTs.^[^ ^126^ ^]^ However, the purity of separated metallic or semiconducting SWCNTs was very low. To understand IEX‐based separation, Lustig et al systematically investigated the effects of the DNA wrapping configuration on the tube dielectric properties by theoretical calculations, proposing an electrostatic interaction‐based mechanism.^[^ ^156^ ^]^ Subsequent studies demonstrated that the hydrophobicity of the SWCNTs and van der Waals interactions between DNA‐wrapped SWCNTs and IEX resin play an important role in IEX‐based separation.^[^ ^101^, ^197^ ^]^ Although the purity of the separated semiconducting and metallic SWCNTs is low in the DNA system, the chiral selectivity of DNA sequences provides an advantage for single‐chirality separation of SWCNTs.

**Figure 8:**
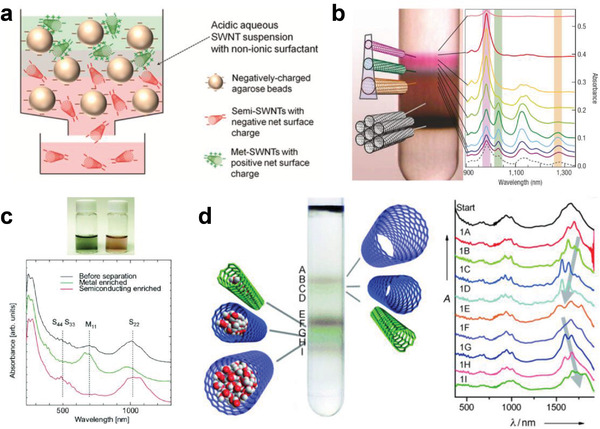
a) Schematic of semiconducting and metallic SWCNT separation based on the charge sign reversal chromatographic technique. a) Reproduced with permission.^[^ ^187^ ^]^ Copyright 2016, American Chemical Society. b) Image of the SWCNTs layered in a centrifugal tube after centrifugation and the optical absorption spectrum of the collected SWCNT solution in each layer. b) Reproduced with permission.^[^ ^99^ ^]^ Copyright 2006, Nature Publishing Group. c) Photographs and optical absorption spectra of separated metallic and semiconducting (right) SWCNT solutions by sucrose‐DGU. c) Reproduced with permission.^[^ ^198^ ^]^ Copyright 2008, American Chemical Society. d) Photograph (left) of arc SWCNTs layered in a centrifuge tube after 24 h of centrifugation and the optical absorption spectrum of the SWCNT solution collected from each layer; the spectra were background‐subtracted, normalized, and offset for clarity. d) Reproduced with permission.^[^ ^199^ ^]^ Copyright 2011, Wiley‐VCH.

In addition to DNA, the nonionic surfactants were later proven to be effective for IEX‐based metallic/semiconducting separation by employing the redox reaction of SWCNTs with an oxygen/water couple at an optimized pH. A schematic of the sorting process is shown in **Figure** **8**a. With this improved technique, the purity of separated semiconducting SWCNTs can reach 99.94% ± 0.04%.^[^ ^187^ ^]^ However, further development of the IEX method has rarely been reported in recent years. As an early developed separation technique, the IEX method has been proven to be very effective for the structure separation of SWCNTs, but the resolution in metallic and semiconducting SWCNTs is relatively low, especially for metallic SWCNTs. Despite this limitation, a large number of successful experiences in the separation of metallic/semiconducting SWCNTs laid an important foundation for subsequent IEX‐based chirality separation described in later sections, which also facilitated the development of other separation techniques.

### 3.3 Density Gradient Centrifugation‐Based Metallic/Semiconducting Separation

DGU is a separation or purification method in which different components can be layered (or separated) in a centrifuge tube with density differences constructed by density gradient agents under centrifugal force. This method has been widely applied in biochemistry for the separation of various subcellular components, such as DNA and protein.^[^ ^200^ ^]^ In 2005, Arnold et al. employed DGU for the diameter‐based separation of DNA‐wrapped SWCNTs.^[^ ^183^ ^]^ 1 year later, they further demonstrated that DGU can also be used to separate SWCNTs dispersed in surfactant solutions of sodium cholate (SC) and sodium deoxycholate (DOC) by their diameter, band gap, and electronic structure (Figure 8b).^[^ ^99^ ^]^ These pioneering works stimulated many follow‐up works on the DGU‐based structure separation of SWCNTs by other groups. Yanagi et al. proposed a DGU approach that included the addition of DOC as a cosurfactant to modulate the interactions between metallic SWCNTs and surfactants, which improved the selectivity of metallic SWCNTs in diameter and thus achieved the separation of metallic SWCNTs of three different colors with a narrow chirality distribution.^[^ ^201^, ^202^ ^]^ Feng et al. developed a self‐generated density gradient method in which a uniform‐density SWCNT dispersion filled into a centrifuge tube. A density gradient was generated by ultracentrifugation, resulting in the separation of metallic and semiconducting SWCNTs, and the purity was comparable to that achieved by DGU using a step‐like density gradient.^[^ ^203^ ^]^ Subsequently, the DGU method was reported to be effective for polymer‐wrapped SWCNTs, showing high extraction efficiency (>65%) and purity (>99%) for both metallic and semiconducting SWCNTs, which is an obvious advantage over surfactant‐based DGU separation.^[^ ^204^, ^205^ ^]^ In addition to dispersants, temperature is an important factor that enables improved separation capability. By lowering the temperature and modulating the concentrations of inexpensive sucrose as a gradient medium that can be easily removed from the SWCNT solution using a centrifuge filter, Yanagi et al. achieved the separation of metallic and semiconducting SWCNTs.^[^ ^198^ ^]^ The purities of the obtained metallic and semiconducting SWCNTs were estimated to be 69% and 95%, respectively, from their optical absorption spectra (Figure 8c), but both values were low by current standards.

An important advantage of the DGU method is that the length‐based separation of SWCNTs can also be achieved simultaneously during the structure separation of SWCNTs through the precise control of the dispersants and processing conditions.^[^ ^206^, ^207^ ^]^ When the bundle‐removed SWCNT solution was ultracentrifuged with a density gradient of iohexol in D_2_O, the water‐filled SWCNTs could be separated from empty SWCNTs (Figure 8d).^[^ ^199^, ^208^, ^209^ ^]^ This progress lays an important foundation for further DGU‐based chirality and enantiomer separation of SWCNTs, as described in later sections. Although DGU enables the excellent structural selectivity for SWCNTs, the complex separation processes, long‐term operation and high‐cost density gradient agents dramatically increase the separation cost and limit the efficiency of separating the semiconducting SWCNTs. Therefore, DGU has not been preferentially used for the structure separation of SWCNTs in recent years, and related work has rarely been reported.

### 3.4 Gel Chromatography‐Based Metallic/Semiconducting Separation

**Figure 9:**
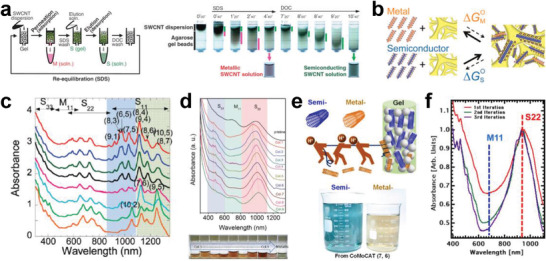
a) Schematic diagram of semiconducting and metallic separation based on the column filled with agarose gel beads (left) and sequential photographs of the gel column and collected SWCNT solutions (right). a) Reproduced with permission.^[^ ^184^ ^]^ Copyright 2009, The Japan Society of Applied Physics. b) The difference in the free energy of adsorption for metallic and semiconducting SWCNTs. b) Reproduced with permission.^[^ ^212^ ^]^ Copyright 2012, American Chemical Society. c) Optical absorption spectra of the separated semiconducting SWCNT solutions by DOC eluents with different concentrations. c) Reproduced with permission.^[^ ^213^ ^]^ Copyright 2010, American Chemical Society. d) Optical absorption spectra of the separated semiconducting solutions from different columns and the pristine SWCNT solution (top). Photographs of the separated semiconducting SWCNT solutions from different columns and the metallic SWCNT solutions (bottom). d) Reproduced with permission.^[^ ^148^ ^]^ Copyright 2017, Wiley‐VCH. e) Schematic (top) of semiconducting and metallic separation by acid regulation and photographs (bottom) of the separated milligram‐scale semiconducting and metallic SWCNT solutions from the CoMoCAT (7,6) material. e) Reproduced with permission.^[^ ^221^ ^]^ Copyright 2018, American Chemical Society. f) Optical absorption spectra of the semiconducting SWCNT solutions after the first, second, and third iterations of separation based on gel chromatography. f) Reproduced with permission.^[^ ^227^ ^]^ Copyright 2013, American Chemical Society.

Gel chromatography is a chromatographic method that employs a gel medium as the stationary phase and has been widely used for the separation of biological macromolecules based on their different retention times in the stationary phase.^[^ ^210^ ^]^ In 2009, Tanaka et al. used gel chromatography to separate metallic and semiconducting SWCNTs.^[^ ^102^ ^]^ They found that the affinity of the agarose gel is significantly stronger for SDS‐wrapped semiconducting SWCNTs than for metallic SWCNTs, so only the semiconducting SWCNTs were adsorbed onto the agarose gel. After the samples were squeezed or centrifuged, the semiconducting SWCNTs were extracted from the agarose gel, resulting in the separation of metallic and semiconducting SWCNTs. To simplify the complicated procedures of extracting semiconducting SWCNTs from gel, the gel chromatography method was improved by using a column packed with gel (**Figure** **9**a).^[^ ^184^ ^]^ After the SWCNT dispersion was injected into the prepared gel column, the SDS‐wrapped metallic SWCNTs directly flowed through the gel column under gravity, and then the semiconducting SWCNTs adsorbed on the gel column were eluted with a DOC eluent. A similar gel column method for the separation of metallic and semiconducting SWCNTs was reported by Kappes et al.^[^ ^211^ ^]^ Their method was different because they used Sephacryl gel as the stationary phase. Newly developed gel chromatography is more suitable for the large‐scale metallic and semiconducting separation of SWCNTs and is still in use today due to its great advantages in simplicity, low cost, and high scalability.

To understand the separation mechanism, Hirano et al. examined the thermodynamics of the adsorption of SWCNTs on gels and attributed the separation of metallic and semiconducting SWCNTs to their difference in the free energy of their adsorption onto gel. The adsorption free energy of semiconducting SWCNTs onto gel is lower than that of metallic SWCNTs (Figure 9b).^[^ ^212^ ^]^ In gel chromatography‐based separation of metallic and semiconducting SWCNTs, many important factors have been gradually found to affect the affinity between SWCNTs and gel, including the solvent component and concentration,^[^ ^29^, ^145^, ^213^, ^214^ ^]^ gel structure and concentration,^[^ ^215^, ^216^, ^217^, ^218^, ^219^ ^]^ pH,^[^ ^148^, ^220^, ^221^ ^]^ temperature,^[^ ^149^, ^212^, ^222^, ^223^, ^224^ ^]^ and separation procedures.^[^ ^225^ ^]^ For example, Liu et al. found that incremental increases in the concentration of DOC in the eluent enables stepwise elution of adsorbed semiconducting SWCNTs with diameters ranging from small to large due to their diameter‐dependent interactions with the gel; this discovery provided an approach to the diameter‐selective separation of semiconducting SWCNTs (Figure 9c).^[^ ^213^ ^]^ Later, modulation of the DOC concentration was also found to be efficient for the purification of metallic SWCNTs.^[^ ^145^ ^]^ In addition to the initial agarose, several kinds of gels, such as the commercialized Sepharose and Sephacryl series and self‐synthesized gels, were found to have the ability to selectively adsorb semiconducting SWCNTs. Similarly, in addition to the initial SDS and DOC, several surfactants, such as SC, SDBS, and their cosurfactants, were also proven to selectively wrap SWCNTs and were suitable for the structure separation of SWCNTs. However, most of the early reports were mainly limited to the metallic and semiconducting separation of SWCNTs with small diameters (<1 nm). To obtain semiconducting SWCNTs with large diameters (>1 nm) due to their higher carrier mobility and lower contact resistance with metal electrodes, it is critical to solve the issue on their weak adsorbability into the gel. In the previous work, the introduction of NaOH or NaCl has been proven to be an effective way to enhance the interaction between small‐diameter SWCNTs and gel.^[^ ^220^, ^226^ ^]^ Recently, Yang et al. achieved the separation of semiconducting SWCNTs with diameters larger than 1.2 nm by NaOH‐assisted gel chromatography (Figure 9d).^[^ ^148^ ^]^ Furthermore, the mass separation of high‐purity semiconducting SWCNTs was recently reported by Cui et al., in which HCl was introduced to selectively oxidize metallic SWCNTs and enlarge the difference in the interaction of metallic and semiconducting SWCNTs with gel (Figure 9e).^[^ ^221^ ^]^ To meet the requirements of practical applications, Tulevski et al. employed iterative gel chromatography separation and successfully improved the purity of semiconducting SWCNTs to 99.9%, which was estimated by a high‐throughput electrical characterization method (Figure 9f).^[^ ^227^ ^]^ Recently, several kinds of gel media with different adsorption sites were synthesized to enhance the adsorption capacity of SWCNTs on the gel, achieving an improvement in the separation efficiency of SWCNTs.^[^ ^215^, ^216^, ^218^, ^219^ ^]^

### 3.5 Aqueous Two‐Phase‐Based Metallic/Semiconducting Separation

**Figure 10:**
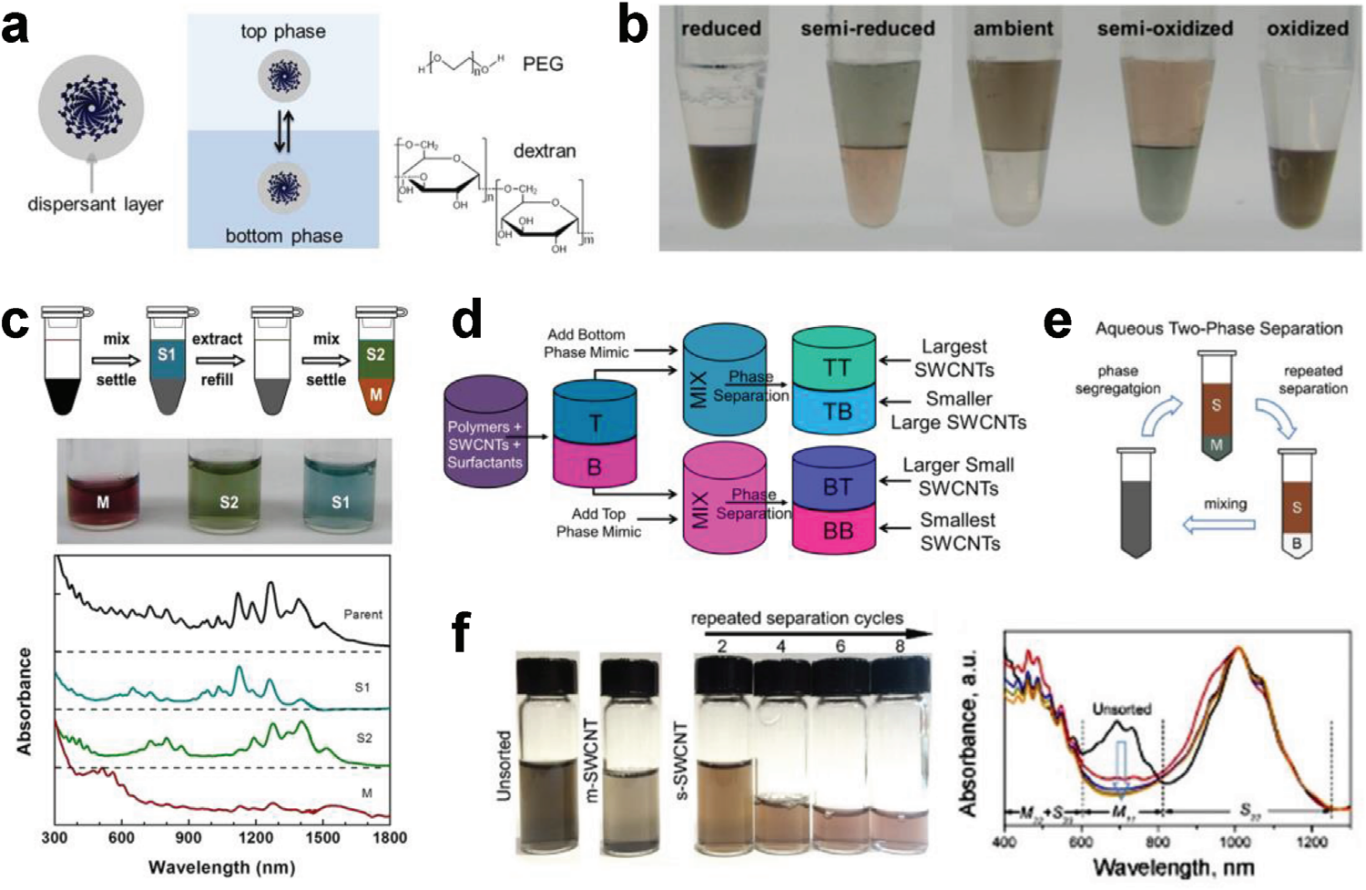
a) Schematic of the ATP method used for the separation of SWCNTs. The top and bottom phases are PEG‐rich and dextran‐rich, respectively. a) Reproduced with permission.^[^ ^103^ ^]^ Copyright 2013, American Chemical Society. b) Redox modulation of nanotube partitioning in a 6% PEG + 6% DX ATO system, where the type and amount of the redox agent added in each case are different. c) Schematic of oxidative extraction, photographs of extracted solutions, and optical absorption spectra of pristine HiPco SWCNTs and extracted semiconducting/metallic SWCNTs. b,c) Reproduced with permission.^[^ ^186^ ^]^ Copyright 2015, American Chemical Society. d) Schematic of the ATP process for diameter separation of SWCNTs. d) Reproduced with permission.^[^ ^229^ ^]^ Copyright 2015, American Chemical Society. e) Schematic of the multiple‐cycle ATP‐based separation procedure, where S and M indicate semiconducting and metallic SWCNT‐enriched phases, respectively, and B indicates the blank bottom phase without SWCNTs. f) Photographs (left) and optical absorption spectra (right) of SWCNT solutions before and after multiple‐cycle separation. e,f) Reproduced with permission.^[^ ^188^ ^]^ Copyright 2017, The Royal Society of Chemistry.

The ATP system means that when two specific polymer aqueous solutions are mixed, the solution will spontaneously form a system composed of a more hydrophobic upper layer and a more hydrophilic lower layer due to their different hydrophilicity and hydrophobicity. As early as thirty years ago, the properties of the ATP system were systematically studied and widely applied to the purification or separation of various cells, proteins, metal nanoparticles, etc.^[^ ^228^ ^]^ In 2013, Zheng's group applied the ATP technique for the structure separation of SWCNTs, in which more hydrophobic polyethylene glycol (PEG) and less hydrophobic dextran (DX) were used as the top and bottom phases, respectively (**Figure** **10**a).^[^ ^103^ ^]^ The separation of metallic and semiconducting SWCNTs was driven by the hydrophobicity of the surfactants adsorbed on the nanotube surface. Because of the selective adsorption of surfactants onto metallic and semiconducting SWCNTs, semiconducting SWCNTs were more hydrophobic and thus selectively partitioned in the top phase, while metallic SWCNTs displaying less hydrophobicity were selectively separated into the bottom phase. This method has attracted great attention due to its advantages of simplicity, low cost, and high scalability.

Subsequently, the redox reactions were found to trigger the reorganization of the surfactant coating layer on the nanotubes, which strongly modulates the nanotube partition in the ATP system (Figure 10b,c).^[^ ^186^ ^]^ Through redox reactions that use NaBH_4_ as the reductant and NaClO as the oxidant, armchair (chiral angle = 30°) and nonarmchair (chiral angle < 30°) metallic SWCNTs were separated.^[^ ^186^ ^]^ In 2015, Fagan et al. achieved an effective separation of metallic and semiconducting SWCNTs with different diameters ranging from 0.6 to 1.7 nm through the multistage ATP method (Figure 10d).^[^ ^229^ ^]^ This methodology was demonstrated to be applicable for multiple commercial SWCNT raw materials, including HiPco, plasma torch, laser vaporization, and arc discharge.^[^ ^229^ ^]^ On the other hand, pH control and multiple‐cycle ATP separation were found to be effective for improving the purity of the separated semiconducting SWCNTs (e.g., higher than 99.5% for semiconducting SWCNTs after four cycles) (Figure 10e,f).^[^ ^188^, ^230^ ^]^

In addition to the surfactant system mentioned above, DNA molecules as dispersants have also proven to be effective for ATP‐based separation. Ao et al. demonstrated the spontaneous partitioning of DNA‐dispersed SWCNTs with higher sensitivity in various two‐phase systems constructed by polyacrylamide, poly(vinylpyrrolidone), PEG, and EX, achieving not only metallic and semiconducting separation but also chirality and enantiomer separation.^[^ ^231^ ^]^ The ATP method has exhibited many advantages, especially high separation resolution, which provides an important foundation for the high‐purity production of SWCNTs with specific structures. However, expensive reagents (DNA and polymer) and multiple separation procedures may hinder the large‐scale separation and applications of SWCNTs in the future. The development of a low‐cost ATP system is highly desirable.

### 3.6 Applications of Semiconducting Single‐Wall Carbon Nanotubes in Electronic Devices

**Figure 11:**
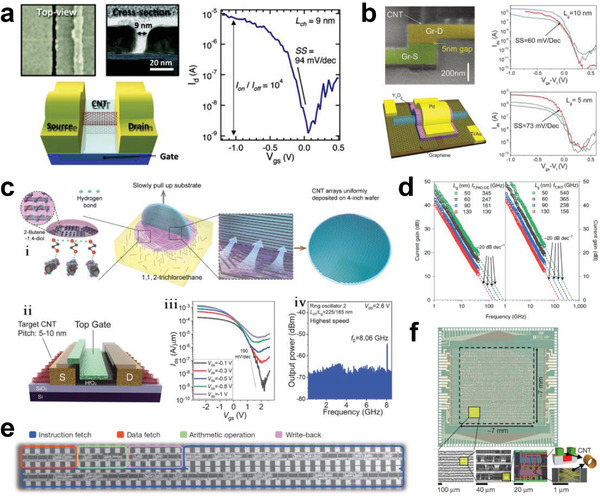
a) Top‐view SEM image, cross‐sectional TEM image, and schematic diagram of the bottom‐gate FET based on SWCNTs (left). The subthreshold characteristics of a SWCNT‐based FET with a channel length of 9 nm. a) Reproduced with permission.^[^ ^237^ ^]^ Copyright 2012, American Chemical Society. b) SEM image of a SWCNT FET with a channel length of 5 nm using graphene as contact electrodes before the deposition of the top gate electrode and the corresponding schematic diagram of the fabricated FET (left). Transfer characteristics of three typical FETs with gate lengths of 10 and 5 nm (right). b) Reproduced with permission.^[^ ^52^ ^]^ Copyright 2017, American Association for the Advancement of Science. c) i) Schematic illustration of the preparation process of a wafer‐scale SWCNT array. ii) Schematic of a SWCNT‐based top‐gate FET with a target SWCNT pitch of 5 to 10 nm. iii) Typical transfer characteristics of SWCNT‐based FETs under various *V*_ds_ voltages. iv) Power spectrum from the champion ring oscillator with the highest switching frequency of 8.06 GHz, corresponding to a stage‐switching speed of 80.6 GHz. c) Reproduced with permission.^[^ ^54^ ^]^ Copyright 2020, American Association for the Advancement of Science. d) Pad de‐embedding (left) and intrinsic (right) current gain versus frequency of SWCNT‐based transistors with gate lengths of 50, 60, 90, and 130 nm. d) Reproduced with permission.^[^ ^241^ ^]^ Copyright 2021, Nature Publishing Group. e) SEM image of an entire carbon nanotube computer. e) Reproduced with permission.^[^ ^243^ ^]^ Copyright 2013, Macmillan Publishers Limited. f) Image of a fabricated RV16X‐NANO chip and local SEM images with different magnification ratios. f) Reproduced with permission.^[^ ^245^ ^]^ Copyright 2019, Nature Publishing Group.

Semiconducting SWCNTs enable good size reduction in device fabrication and have extremely high carrier mobility, tunable direct bandgaps, and solution processability, making them ideal materials for high‐performance electronic and optoelectronic devices and ICs.^[^ ^14^, ^15^, ^16^, ^19^, ^20^, ^21^, ^22^, ^25^, ^26^, ^27^ ^]^ Furthermore, because of their excellent mechanical properties and environmental stability, semiconducting SWCNTs are ideal material for the fabrication of flexible electronic devices used in various extreme environments.^[^ ^232^, ^233^, ^234^, ^235^, ^236^ ^]^ In 2012, Franklin et al. constructed the first sub‐10 nm SWCNT transistor from IBM. It can operate quickly (94 mV/decade for subthreshold slope) at a low operating voltage (0.5 V), and it was better than silicon devices of similar dimensions and exhibited better scaling behavior than theoretical prediction (**Figure** **11**a).^[^ ^237^ ^]^ In 2017, Peng's group scaled down the size of SWCNT‐based transistors to a gate length of 5 nm by using graphene as the contact electrode (Figure 11b).^[^ ^52^ ^]^ This smallest SWCNT transistor can operate much faster (73 and 60 mV/decade for gate lengths of 5 and 10 nm, respectively) at much lower operation voltages (0.2 V) than previous resistors, showing higher performance than an Si complementary metal‐oxide semiconductor at the same scale. These results clearly demonstrated that the semiconducting SWCNT material has emerged as an important candidate for future electronic devices.

With the advancement in the bulk separation of semiconducting SWCNTs, researchers have started to focus on the fabrication of high‐performance TFTs. As early as 2006, Arnold et al. constructed SWCNT‐based TFTs using semiconducting SWCNTs with purities higher than >97% separated by DGU,^[^ ^99^ ^]^ which showed an on/off ratio larger than 10^4^. Lei et al. fabricated TFTs with channel lengths ranging from 5 to 30 µm using semiconducting SWCNTs with purities higher than 99.7% separated by a polymer wrapping method.^[^ ^193^ ^]^ Among these TFTs, devices with channel lengths >10 µm exhibited hole mobilities in the range of 20–49 cm^2^ V^−1^ s^−1^ with an on/off ratio >10^6^.^[^ ^193^ ^]^ A higher carrier mobility (164 cm^2^ V^−1^ s^−1^) was achieved by using length‐sorted semiconducting SWCNTs.^[^ ^167^, ^168^ ^]^ After further controlling the nanotube orientation on the substrate, the on/off ratio increased to 10^7^–10^8^.^[^ ^168^, ^238^, ^239^ ^]^ Most recently, Arnold's group fabricated high‐performance TFTs by removing the polymers around semiconducting SWCNTs separated by the polymer wrapping method, and their performance exceeded those made of traditional semiconductor materials.^[^ ^240^ ^]^ Peng's group employed ultrahigh‐purity (>99.9999%) semiconducting SWCNTs separated by a newly developed multiple dispersion and separation process to prepare high‐density well‐aligned arrays (Figure 11c) and then fabricated TFTs and ICs.^[^ ^54^ ^]^ These devices display an on‐state current of 1.3 mA µm^−1^ and oscillating frequency of >8 GHz, exceeding the performances of commercial silicon‐based devices with similar device sizes.^[^ ^54^ ^]^ Furthermore, Peng's group constructed SWCNT array‐based FETs with a 50 nm gate length, which exhibited current‐gain and power‐gain cutoff frequencies of 540 and 306 GHz, respectively (Figure 11d).^[^ ^241^ ^]^ These results experimentally validate a promising path toward practical electronic systems that do not require silicon.

In addition to the preparation of SWCNT‐FETs, researchers have also focused on SWCNT‐based applications in terms of large‐scale ICs using sorted semiconducting SWCNTs.^[^ ^54^, ^235^, ^236^, ^242^, ^243^, ^244^, ^245^, ^246^, ^247^, ^248^, ^249^, ^250^ ^]^ In 2013, Shulaker et al. used semiconducting SWCNTs with a purity higher than 99.99% to build the first carbon nanotube computer, which enables counting and integer sorting simultaneously and is the most complex carbon‐based electronic system reported thus far (Figure 11e).^[^ ^243^ ^]^ After that, they demonstrated SWCNT‐based 3D integration for computing and data storage on a single chip.^[^ ^251^ ^]^ Recently, they further fabricated a 16‐bit microprocessor composed of more than 14 000 SWCNT‐based FETs, which successfully runs standard 32‐bit instructions on 16‐bit data and addresses (Figure 11f).^[^ ^245^ ^]^ More importantly, this microprocessor was designed and fabricated by using current industry‐standard design flows and processes, and showed the good compatibility of SWCNT materials and Si‐based semiconductor processes. Current SWCNT‐based electronic devices are mainly made of semiconducting SWCNTs containing various chiral species with different energy bands, which inevitably results in the unpredictable performances of the prepared devices. Therefore, mass preparation of single‐chirality semiconducting species is a prerequisite for the development of high‐performance SWCNT‐based electronic and optoelectronic ICs.

## 4 Separation of Single‐Chirality Single‐Wall Carbon Nanotubes and Their Applications

**Figure 12:**
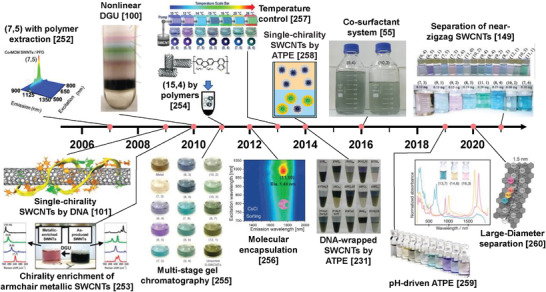
Timeline of separation of single‐chirality SWCNTs. Reproduced with permission from the following: Reproduced with permission.^[^ ^252^ ^]^ Copyright 2007, American Chemical Society; Reproduced with permission.^[^ ^101^ ^]^ Copyright 2009, Nature Publishing Group; Reproduced with permission.^[^ ^253^ ^]^ Copyright 2010, American Chemical Society; Reproduced with permission.^[^ ^100^ ^]^ Copyright 2010, Nature Publishing Group; Reproduced with permission.^[^ ^254^ ^]^ Copyright 2011, American Chemical Society; Reproduced with permission.^[^ ^255^ ^]^ Copyright 2011, Nature Publishing Group; Reproduced with permission.^[^ ^256^ ^]^ Copyright 2012, American Chemical Society; Reproduced with permission.^[^ ^257^ ^]^ Copyright 2013, American Chemical Society; Reproduced with permission.^[^ ^258^ ^]^ Copyright 2014, Wiley‐VCH; Reproduced with permission.^[^ ^231^ ^]^ Copyright 2014, American Chemical Society; Reproduced with permission.^[^ ^55^ ^]^ Copyright 2016, Nature Publishing Group; Reproduced with permission.^[^ ^259^ ^]^ Copyright 2019, American Chemical Society; Reproduced with permission.^[^ ^260^ ^]^ Copyright 2019, American Chemical Society; Reproduced with permission.^[^ ^149^ ^]^ Copyright 2019, American Association for the Advancement of Science.

Compared with the separation of metallic and semiconducting SWCNTs, the separation of single‐chirality SWCNTs faces greater challenges due to the requirement of higher‐resolution separation techniques. Based on the metallic and semiconducting separation of SWCNTs, scientists have been further working to develop various techniques for the separation of chiral structures of SWCNTs, including polymer wrapping, IEX, DGU, gel chromatography, and ATP (**Figure** **12**). In this section, we provide a systematic review of recent advances in the separation of single‐chirality SWCNTs with these techniques. Because the chirality resolution of each separation method is different, the purity and quantity of single‐chirality SWCNTs obtained by different methods are quite different. The details are described in the following sections.

### 4.1 Polymer Wrapping‐Based Chirality Separation

**Figure 13:**
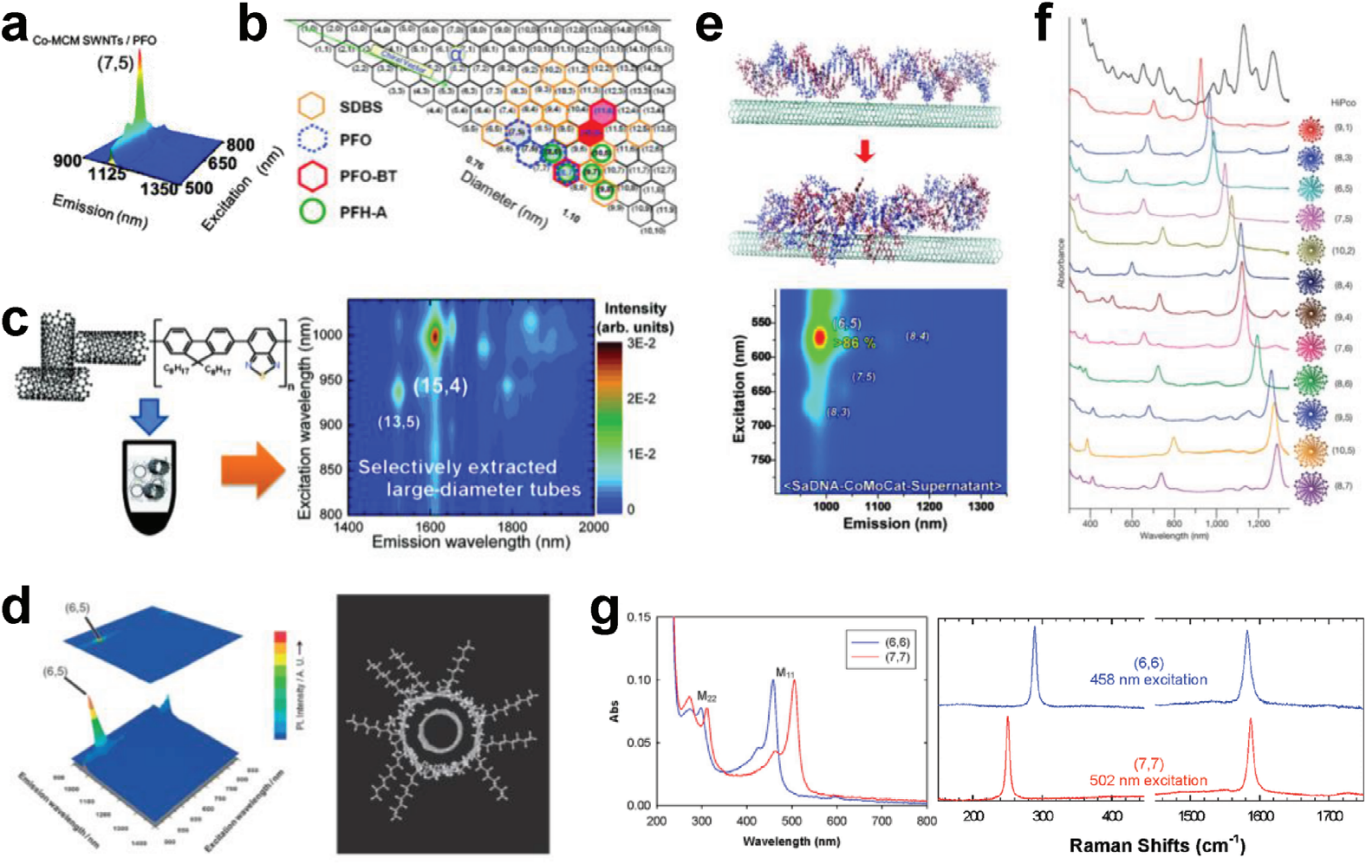
a) PL excitation–emission map of single‐chirality (7, 5) SWCNTs separated by the PFO‐based polymer wrapping method. b) Chirality map of SWCNTs wrapped by SDBS, PFO, PFO‐BT, and PFH‐A. a,b) Reproduced with permission.^[^ ^252^ ^]^ Copyright 2007, American Chemical Society. c) Schematic of large‐diameter semiconducting SWCNT separation using fluorene‐based polymers and the PL excitation–emission map of separated (15, 4) SWCNTs. c) Reproduced with permission.^[^ ^254^ ^]^ Copyright 2011, American Chemical Society. d) PL excitation–emission map of (6, 5) SWCNTs solubilized by PFO‐BPy in p‐xylene (left) and the optimized model structures of a (6, 5) SWCNT wrapped by the trimer of 9,9‐dioctylfluorenyl‐2,7‐diylBPy (right). d) Reproduced with permission.^[^ ^165^ ^]^ Copyright 2011, The Chemical Society of Japan. e) Transitions of conformations from native SaDNA to an energy‐minimized DNA‐SWCNT hybrid obtained from classical all‐atom MD simulations. PL excitation–emission map of SaDNA dispersed CoMoCat SWCNTs after ultracentrifugation. e) Reproduced with permission.^[^ ^264^ ^]^ Copyright 2008, American Chemical Society. f) Optical absorption spectra of 12 kinds of separated single‐chirality SWCNTs and the pristine HiPco mixture, accompanied by the atomic structure of each chirality species. f) Reproduced with permission.^[^ ^101^ ^]^ Copyright 2009, Macmillan Publishers Limited. g) Optical absorption (left) and Raman spectra (right) of the separated single‐chirality (6, 6) and (7, 7) armchair SWCNTs wrapped by DNA. g) Reproduced with permission.^[^ ^266^ ^]^ Copyright 2011, American Chemical Society.

As mentioned above, the separation of high‐purity semiconducting SWCNTs was realized by the selective coating of polymers on their surfaces. In addition, early reports have demonstrated that certain polymers have obvious chirality selectivity and can disperse semiconducting SWCNTs if their molecular structures are appropriately designed, revealing the possibility of using these polymers to separate single‐chirality SWCNTs.^[^ ^98^, ^165^, ^204^, ^252^, ^254^, ^261^, ^262^ ^]^ For example, Chen et al. reported that (7, 5) SWCNTs with a diameter of 0.8 nm can be obtained by polymer‐assisted separation from raw SWCNT materials with a narrow‐diameter distribution (**Figure** **13**a,b).^[^ ^252^ ^]^ The separation of large‐diameter (15, 4) SWCNTs (1.35 nm) was also reported by Tange et al., who used the polymer of poly(9,9‐dioctylfluorene‐alt‐benzothiadiazole), which enables the selective dispersion of (15, 4) SWCNTs (Figure 13c).^[^ ^254^ ^]^ The comparative experiment showed that this selective dispersion is not successful with other polymers or surfactants. Generally, other separation methods cannot be used for the chirality separation of SWCNTs with such large diameters. However, except for (6, 5) species that was separated by Nakashima et al. using bipyridine and 9,9‐dioctylfluorenyl‐2,7‐diyl as extracting agents and m‐xylene and p‐xylene as solvents (Figure 13d),^[^ ^165^ ^]^ the purities of other separated chirality species, such as (7, 5), (7, 6), (9, 4), (9, 5), (10, 5), and (10, 8) SWCNTs, were not high enough at less than 90%.^[^ ^204^, ^254^, ^261^, ^262^ ^]^ Additionally, except for the large‐scale separation of (6, 5) SWCNTs, mass production of other single‐chirality species has not been reported.

The selectivity of polymers is significantly lower for chirality species than for electrical type of SWCNTs. Moreover, the chirality selectivity of polymers is also significantly lower than that of DNA and surfactants. Although the detailed reason is still unclear due to the lack of mechanism studies on the interactions between polymers and distinct chirality species of SWCNTs, the design of the specific polymer structure is considered to be a critical issue in the polymer‐based chirality separation of SWCNTs. In addition to this limitation, the high cost of polymers still limits the industrial production of single‐chirality SWCNTs. Most recently, Flavel et al. estimated the cost of (6, 5) SWCNTs separated by polymer wrapping and ATP methods based on the prices of raw SWCNTs, dispersants (polymers or surfactant), solvents and additives from commercial chemical suppliers.^[^ ^263^ ^]^ The estimated cost of (6, 5) SWCNTs produced by the polymer wrapping method under laboratory conditions is as high as €36 000–73 000 g^−1^, which is much higher than that by ATP (€8300–13 000 g^−1^).^[^ ^263^ ^]^ The separation cost of SWCNTs by gel chromatography is similar to that of ATP. The cost difference between the two methods mainly lies in the difference between gel media and two‐phase media. Although the gel used in gel chromatography is relatively expensive, it can be reused many times, which greatly reduces the separation cost of SWCNTs.^[^ ^35^ ^]^ Nevertheless, it is expected that such high cost under laboratory conditions may be reduced with the optimization of synthesis/separation process and the expansion of production scale in the future SWCNT industry.

### 4.2 Ion‐Exchange Chromatography‐Based Chirality Separation

In 2005, Zheng et al. developed an effective length‐control method based on size exclusion chromatography to obtain SWCNTs with a narrow length distribution,^[^ ^155^ ^]^ avoiding the influence of nanotube length and improving the resolution in the subsequent chirality separation based on IEX. As a result, three kinds of near single‐chirality SWCNTs with similar diameters, (6, 4), (9, 1), and (6, 5), were successfully separated.^[^ ^157^ ^]^ A similar result for (6, 5) SWCNT separation was later achieved by using genomic salmon DNA instead of single‐stranded DNA (ssDNA) adopted in the previous IEX (Figure 13e).^[^ ^264^ ^]^ However, the chirality purities of the obtained SWCNTs were very limited in these early works and were lower than 90%.^[^ ^155^, ^157^, ^264^, ^265^ ^]^

In 2009, another pioneering study was completed by Zheng et al. Through the selection of specific DNA sequences from the vast ssDNA library for IEX separation, they successfully realized the high‐purity separation of 12 distinct single‐chirality species of semiconducting SWCNTs (Figure 13f).^[^ ^101^ ^]^ The chirality purity of each species was much improved (>90%) compared with the previous results from various separation techniques due to the sequence‐ and chirality‐dependent interactions between the DNA and SWCNTs. Subsequently, DNA‐based IEX separation was extended to the chirality separation of armchair metallic SWCNTs by designing new DNA sequences with exquisite sensitivity for metallic SWCNTs (Figure 13g).^[^ ^266^ ^]^ Although the DNA‐based IEX technique has exhibited excellent atomic‐scale recognition for both semiconducting and metallic SWCNTs, using it to produce single‐chirality SWCNTs on a large scale is difficult due to the high cost of DNA and the complex separation processes. Therefore, the IEX technique has not been further developed or optimized for the practical applications of chirality‐sorted SWCNTs.

### 4.3 Density Gradient Centrifugation‐Based Chirality Separation

**Figure 14:**
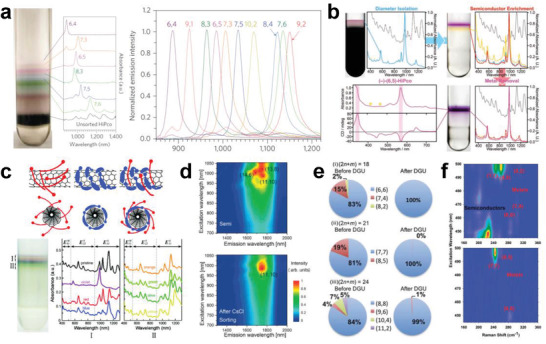
a) Photograph of the SWCNTs layered in a centrifugal tube after an 18‐h nonlinear DGU run (left) and the optical absorption spectra of collected SWCNT solutions from different layers (middle). PL spectra of 10 chirality‐separated solutions excited at the E_22_ wavelength of each chirality species (right). a) Reproduced with permission.^[^ ^100^ ^]^ Copyright 2010, Nature Publishing Group. b) Optical absorption and CD characterization of the separated single‐chirality (6, 5) SWCNTs and their enantiomers from HiPco SWCNTs by using multiple orthogonal DGU iterations involving diameter isolation, semiconductor enrichment, and metal removal. b) Reproduced with permission.^[^ ^267^ ^]^ Copyright 2011, Wiley‐VCH. c) Schematic of the morphology of surfactant SDS only (thin, red shape), DOC only (thick, blue shape), and DOC followed by SDS wrapping on a (6, 5) SWCNT (top), photograph and optical absorption spectra of layered SWCNTs (bottom). c) Reproduced with permission.^[^ ^268^ ^]^ Copyright 2010, American Chemical Society. d) PL excitation–emission maps of separated large‐diameter semiconducting (top) and single‐chirality (11, 10) SWCNTs (bottom). d) Reproduced with permission.^[^ ^256^ ^]^ Copyright 2012, American Chemical Society. e) Relative abundances of armchair species before and after DGU separation evaluated by using resonant Raman scattering. e) Reproduced with permission.^[^ ^269^ ^]^ Copyright 2013, Royal Society of Chemistry. f) Resonant Raman scattering excitation maps of metallic SWCNTs before (top) and after (bottom) chirality enrichment. f) Reproduced with permission.^[^ ^253^ ^]^ Copyright 2010, American Chemical Society.

The key to the separation of single‐chirality SWCNTs by DGU is adjusting their density difference and moving them to different density‐dependent positions in centrifuge tubes under ultracentrifugal forces so that they are separated along the vertical direction of the centrifugal tube. In 2010, Ghosh et al. developed nonlinear density gradients to amplify the differences in their buoyant densities and achieved the simultaneous separation of single chirality and their enantiomers.^[^ ^100^ ^]^ With this technique, ten different single‐chirality species were separated (**Figure** **14**a),^[^ ^100^ ^]^ although the purities of some species were not very high. Subsequently, single‐chirality separation of (6, 5) SWCNTs was also achieved by Green et al. via orthogonal iterative DGU (Figure 14b).^[^ ^267^ ^]^ Zhao et al. proposed cosurfactants of SDS and DOC to selectively interact with distinct chirality species and to amplify the differences in their buoyant densities, which led to the efficient chirality redistribution and the separation of (6, 5) SWCNTs with a chiral purity of 97% (Figure 14c).^[^ ^268^ ^]^ Yanagi et al. amplified the density differences of different SWCNTs by the selective encapsulation of organic molecules and achieved the single‐chirality separation of (11, 10) SWCNTs (Figure 14d).^[^ ^256^ ^]^

In addition to semiconducting SWCNTs, the DGU method also shows high resolution for the chiral structures of metallic SWCNTs. In 2010, Kono et al. achieved the chirality enrichment of armchair and near‐armchair metallic SWCNTs after optimization under sample dispersion conditions and fractionation.^[^ ^253^ ^]^ The high enrichment of metallic SWCNTs was determined by resonant Raman scattering (Figure 14e,f).^[^ ^253^, ^269^ ^]^ Due to the successful separation of single‐chirality metallic SWCNTs, the unique coloration mechanism and electrochromic modulation of 1D metallic SWCNTs were subsequently clarified.^[^ ^270^, ^271^ ^]^ These results clearly demonstrated the excellent structural selectivity of the DGU method, but the expensive density gradient medium, time‐consuming separation process and tedious collection limited the industrial production of single‐chirality species, and as such, DGU‐based single‐chirality separation has rarely been reported in recent years.

### 4.4 Gel Chromatography‐Based Chirality Separation

**Figure 15:**
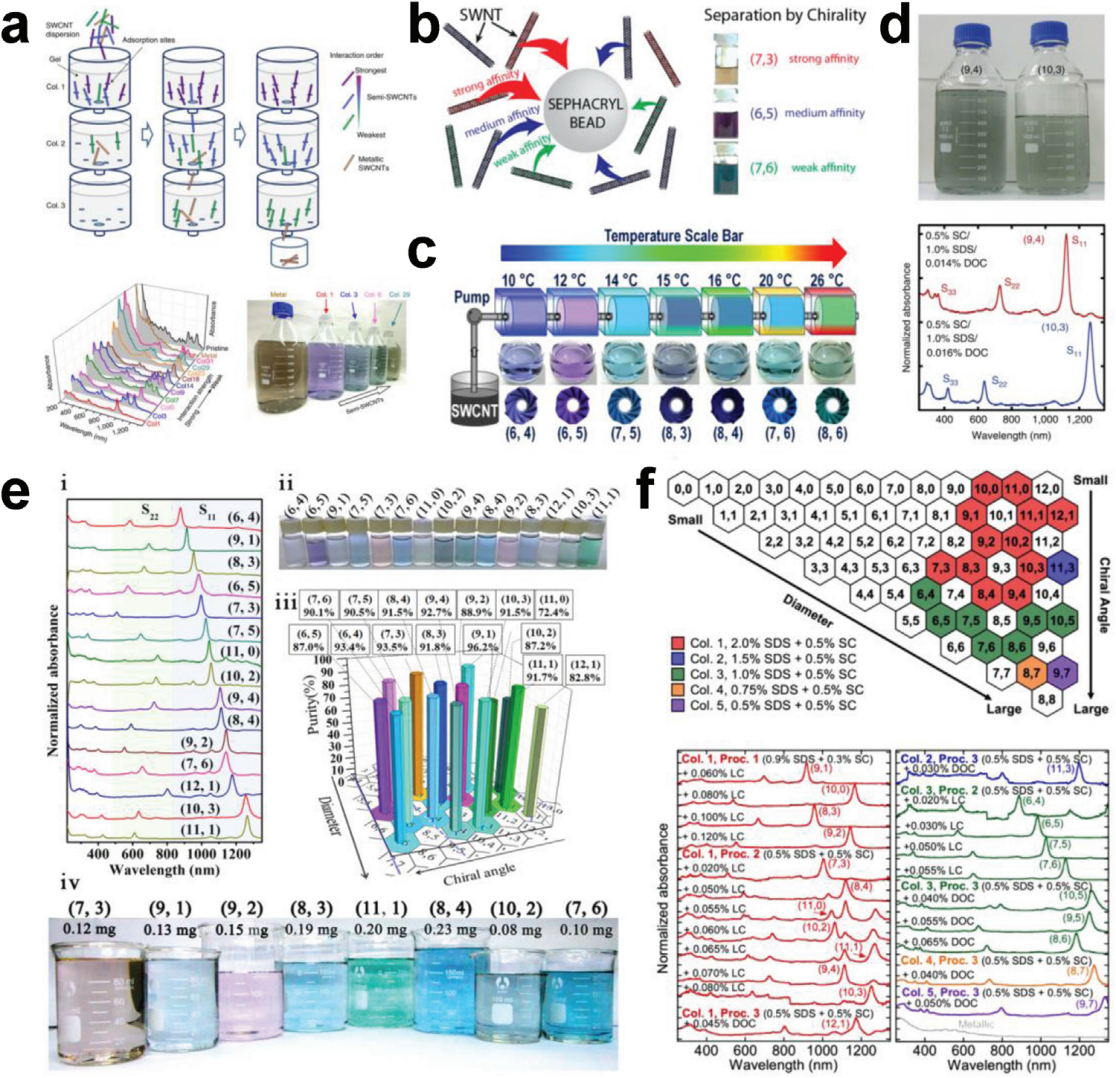
a) Schematic of competitive adsorption of semiconducting SWCNTs with different chirality species in multicolumn gel chromatography (top), optical absorption spectra, and photograph of separated metallic and semiconducting SWCNT solutions. a) Reproduced with permission.^[^ ^255^ ^]^ Copyright 2011, Macmillan Publishers Limited. b) Schematic of the kinetically driven competitive binding model for single‐chirality separation of semiconducting SWCNTs in gel chromatography. b) Reproduced with permission.^[^ ^272^ ^]^ Copyright 2013, American Chemical Society. c) Schematic of temperature‐controlled gel chromatography for high‐efficiency single‐chirality separation of SWCNTs. c) Reproduced with permission.^[^ ^257^ ^]^ Copyright 2013, American Chemical Society. d) Photographs (top) and optical absorption spectra (bottom) of separated milligram‐scale (9, 4) and (10, 3) SWCNTs. d) Reproduced with permission.^[^ ^55^ ^]^ Copyright 2016, Nature Publishing Group. e) i) Optical absorption spectra of 15 single‐chirality SWCNTs separated by temperature‐controlled gel chromatography. ii) Photographs of the separated single‐chirality SWCNT solutions. iii) Purity distribution of each separated single‐chirality SWCNTs evaluated by optical spectroscopy. iv) Photograph of the obtained submilligram‐scale single‐chirality near‐zigzag SWCNTs. e) Reproduced with permission.^[^ ^149^ ^]^ Copyright 2021, American Association for the Advancement of Science. f) Chirality map of SWCNTs separated using five columns. Each column corresponds to the mixed surfactants, including SDS and SC, with different concentrations (top). Optical absorption spectra of high‐purity (*n*, *m*) species eluted by multiple processes in each column corresponding to the mixed surfactants of SDS + SC + DOC or SDS + SC + LC (bottom). f) Reproduced with permission.^[^ ^278^ ^]^ Copyright 2020, American Chemical Society.

After great success in the metallic/semiconducting separation of SWCNTs via the gel chromatography method, the separation of single‐chirality SWCNTs should be further explored. In 2011, Liu et al. developed multi‐stage gel chromatography for the separation of single‐chirality SWCNTs (**Figure** **15**a), in which 13 distinct (*n*, *m*) single‐chirality SWCNTs were produced by their competitive adsorption onto gel medium under overloading.^[^ ^255^ ^]^ They attributed the chirality‐dependent interaction of SWCNTs with allyl dextran‐based gel (Sephacryl S‐200) to the different SDS coverage. Subsequently, this mechanism was further confirmed by the kinetic model proposed by Tvrdy et al. (Figure 15b).^[^ ^272^ ^]^ However, a repetitive overloading process is required to achieve single‐chirality separation, which limits the separation yield and increases the separation cost.

In the following years, many other factors, such as surfactant type and concentration,^[^ ^40^, ^55^, ^273^, ^274^, ^275^, ^276^, ^277^, ^278^, ^279^ ^]^ pH,^[^ ^280^ ^]^ temperature,^[^ ^149^, ^257^ ^]^ gel structure,^[^ ^216^, ^219^ ^]^ and separation procedure,^[^ ^225^, ^281^ ^]^ were employed to enhance the selective adsorption of surfactant molecules and improve the purity, species, and yield of separable single‐chirality SWCNTs. Liu et al. used the temperature to finely tune the SDS coverage on nanotube surfaces, amplifying the difference in the adsorption ability of distinct chirality species onto gels.^[^ ^257^ ^]^ With this technique, a variety of single‐chirality SWCNTs were separated in one step (Figure 15c).^[^ ^257^ ^]^ In particular, the separation yields of (6, 4) and (6, 5) SWCNTs were increased to the submilligram scale. To further improve the resolution of the chiral structures of SWCNTs, the selective interaction of different surfactant molecules with SWCNTs was systematically investigated. On this basis, a cosurfactant system was designed to achieve high‐resolution separation of single‐chirality SWCNTs by selective adsorption and elution in chiral angle and diameter. With this improved gel chromatography, Yomogida et al. realized the separation of single‐chirality (9, 4) and (10, 3) SWCNTs on a large scale (Figure 15d).^[^ ^55^ ^]^ Despite this success, the separable types of SWCNTs that can be separated at a large scale are still very limited, especially for zigzag and near‐zigzag chirality species, and this limitation will inevitably hinder the further study of the properties and applications of SWCNTs. Because of the symmetrical structures of these SWCNTs, it is more suitable to prepare a single photon source by covalent modification. To produce near‐zigzag single‐chirality SWCNTs, Yang et al. developed an approach to separate SWCNTs by chiral angle through temperature control of a binary surfactant system of SC and SDS, in which highly selective adsorption of SWCNTs with a small chiral angle was achieved at temperatures less than 18 °C (Figure 15e).^[^ ^149^ ^]^ Combined with diameter separation, more than 10 types of single‐chirality species, including near‐zigzag (9, 1), (9, 2), (10, 2), and (11, 1) SWCNTs, were successfully separated on the submilligram scale.^[^ ^149^ ^]^ These results indicate that gel chromatography has obvious advantages in the purity, species, and quantity of separable single‐chirality SWCNTs over other separation methods. Most recently, separable single‐chirality SWCNTs by gel chromatography were further expanded to twenty types by introducing a new surfactant of hydrophobic cholate with ultrahigh chirality resolution into the mixed‐surfactant system (Figure 15f).^[^ ^278^ ^]^

In addition to semiconducting SWCNTs, chirality separation of metallic SWCNTs such as (10, 4) was also achieved using a long gel column.^[^ ^273^ ^]^ However, the purities are relatively low, mainly due to the inability of adsorption onto gel. In principle, there are two pathways to enhance the adsorption capacity of metallic SWCNTs on gels: A stationary phase and a mobile phase. In other words, by designing new gel and dispersant compositions with specific structures, it is possible to achieve a strong interaction between metallic SWCNTs and the gels. After that, through the precise modulation of the difference in the interaction of various (*n*, *m*) SWCNTs with gel by surfactant type and concentration, pH, temperature, gel structure, separation procedure, etc., high‐resolution chirality recognition of metallic SWCNTs should be possible.

### 4.5 Aqueous Two‐Phase‐Based Chirality Separation

**Figure 16:**
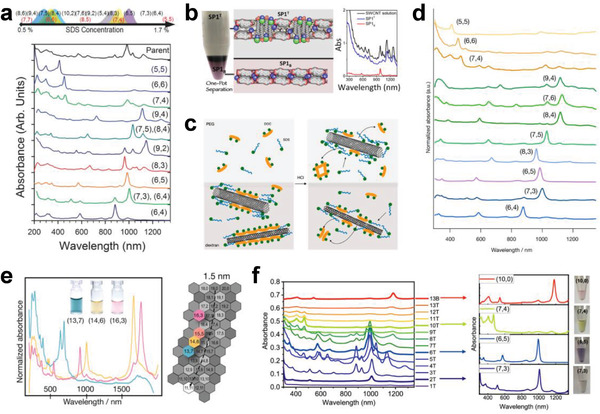
a) Partition order of individual chirality species to the top phase with an increase in SDS concentration (top) and the optical absorbance spectra of chirality species separated by the iterative ATP method. a) Reproduced with permission.^[^ ^258^ ^]^ Copyright 2014, Wiley‐VCH. b) Schematic of the one‐pot ATP separation for controlling the surfactant coverage and the optical absorption spectra before and after separation in only 1 or 2 steps. b) Reproduced with permission.^[^ ^282^ ^]^ Copyright 2014, American Chemical Society. c) Proposed schematic of the mixed surfactants of SDS and DOC wrapped on small‐ and large‐diameter nanotubes before and after the addition of HCl. d) Optical absorption spectra of single‐chirality SWCNT solutions separated by a pH‐controlled ATP method. c,d) Reproduced with permission.^[^ ^259^ ^]^ Copyright 2019, American Chemical Society. e) Optical absorption spectra and chirality map of separated single‐chirality SWCNTs with a diameter of ≈1.41 nm by using the ATP method. e) Reproduced with permission.^[^ ^260^ ^]^ Copyright 2019, American Chemical Society. f) Continuous multistep ATP separation of 4 single‐chirality SWCNTs from a single DNA/SWCNT dispersion. f) Reproduced with permission.^[^ ^286^ ^]^ Copyright 2019, American Chemical Society.

ATP is the fastest‐developing method for the structure separation of SWCNTs. The chirality separation of SWCNTs was achieved only one year after its establishment for the separation of metallic and semiconducting SWCNTs.^[^ ^103^ ^]^ In 2014, Fagan et al. found that the concentration of cosurfactants greatly affects the partitioning of various (*n*, *m*) SWCNTs from the bottom DX phase to the top PEG phase.^[^ ^258^ ^]^ They attribute this phenomenon to the change in the coverage of cosurfactants on the SWCNTs, which enhances the difference in the hydrophobicity of surfactant‐dispersed SWCNTs with different (*n*, *m*) species and thus improves the separation resolution. As shown in **Figure** **16**a, multiple single‐chirality SWCNTs, including metallic and semiconducting SWCNTs, were sequentially separated by gradually increasing the concentration of SDS or DOC in the cosurfactants.^[^ ^258^ ^]^ However, multiple separation steps must be performed to obtain these single‐chirality species. The simplified separation steps could achieve the separation of single‐chirality SWCNTs, but the separable species was limited (Figure 16b).^[^ ^282^, ^283^ ^]^ Recently, Li et al. further finely tuned the coating coverage of cosurfactants on different (*n*, *m*) SWCNTs by precisely controlling the pH of the SWCNT suspension, thus improving the chirality separation resolution (Figure 16c).^[^ ^259^ ^]^ Through this approach, 11 kinds of single‐chirality SWCNTs, including metallic SWCNTs, were successfully separated in no more than three steps (Figure 16d),^[^ ^259^ ^]^ simplifying the ATP separation procedure and demonstrating the ATP‐based large‐scale separation of single‐chirality SWCNTs. In addition, the temperature and the concentration of the PEG polymer were also optimized to improve the separation efficiency of the ATP method.^[^ ^284^ ^]^ Most recently, Zheng et al. realized the single‐chirality separation of large‐diameter SWCNTs, including (8, 8), (9, 9), (10, 10), (13, 7) metallic SWCNTs and (14, 6), (16, 3) semiconducting species, by molecule filling (Figure 16e).^[^ ^229^, ^260^ ^]^

The ATP method enables to separate various single‐chirality not only for the surfactant‐dispersed SWCNTs, but also for the DNA‐dispersed SWCNTs. The first work on the ATP‐based chirality separation of DNA‐dispersed SWCNTs was reported by Zheng's group in 2014.^[^ ^231^ ^]^ They found that the partitioning of DNA‐SWCNT hybrids in a polymer‐modified two‐phase system is strongly dependent on the DNA sequence and polymer composition, which is possibly related to the solvation energy of DNA‐SWCNT hybrids. With the proper combination of DNA sequence, polymer composition, and partition modulators, 15 kinds of single‐chirality SWCNTs were effectively separated from a synthetic CoMoCAT mixture.^[^ ^231^ ^]^ In 2016, the types of separable single‐chirality SWCNTs were expanded to more than 20 as over 300 short ssDNA sequences were screened.^[^ ^285^ ^]^ These chirality species covered the entire chiral angle range and all three electronic classifications (metal, quasi‐metal, and semiconductor), indicating complete separation of the CoMoCAT material. Although the DNA system has a high resolution for the structure of SWCNTs, the separation of each type of SWCNT requires a specific DNA molecule, which inevitably increases the number of steps and the cost of separation and hinders their mass preparation. To address this issue, most recently, Lyu et al. developed a new ATP system of PEG/DX with certain molecular weights (1.5 and 250 kDa for PEG and DX phases, respectively) and realized the separation of 4 chirality species by a single DNA‐SWCNT system (Figure 16f).^[^ ^286^ ^]^ These results indicate that it is also possible to achieve high‐throughput separation of single‐chirality SWCNTs through a single DNA system in the future. It is believed that in the near future, the mass separation of various single‐chirality SWCNTs by ATP may be realized due to its high separation efficiency.

### 4.6 Applications of Single‐Chirality Single‐Wall Carbon Nanotubes in Optoelectronic Devices and Bioimaging

Because of their identical photoelectric properties, single‐chirality SWCNTs exhibit great application prospects in photoelectric devices and biomedicine. Based on the great progress made in separation techniques thus far, the large‐scale separation of high‐purity single‐chirality SWCNT materials is achievable, which lays an important foundation for the development of next‐generation optoelectronic devices. In this section, we will provide a systematic review of recent advances in the typical applications of chirality‐separated semiconducting SWCNTs, including light emission and detection devices and biological imaging.

**Figure 17:**
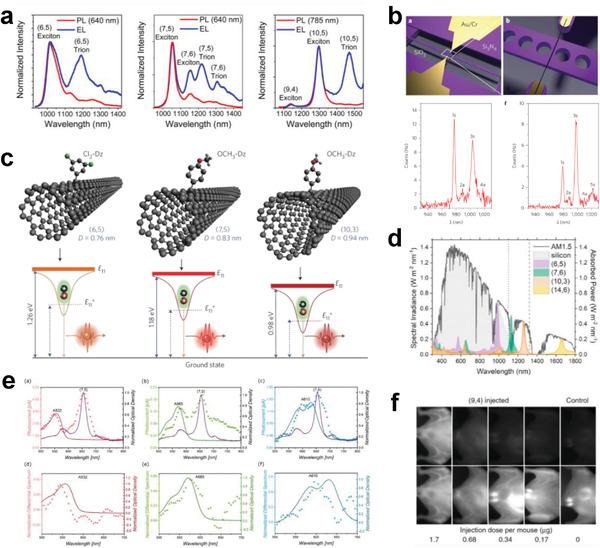
a) PL and EL spectra of electrolyte‐gated transistors based on (6, 5), (7, 5), and (10, 5) SWCNTs. a) Reproduced with permission.^[^ ^290^ ^]^ Copyright 2014, American Chemical Society. b) Schematic of photonic crystal nanobeam cavity device with coupled SWCNT light emitter (top) and the spectra of electrically driven SWCNTs measured at grating couplers for two devices (bottom). b) Reproduced with permission.^[^ ^294^ ^]^ Copyright 2016, Nature Publishing Group. c) Schematic of the aryl‐functionalized (6, 5), (7, 5), and (10, 3) SWCNTs (top) and the corresponding band diagrams (bottom). c) Reproduced with permission.^[^ ^308^ ^]^ Copyright 2017, Nature Publishing Group. d) Spectral overlap of silicon and various (*n*, *m*) SWCNTs with the AM1.5G solar irradiance spectrum. d) Reproduced with permission.^[^ ^340^ ^]^ Copyright 2021, Elsevier Ltd. e) Photocurrent (top) and differential (bottom) spectra of (7, 5) SWCNT devices modified with A532 (left), A565 (middle), and A610 (right) dyes. e) Reproduced with permission.^[^ ^345^ ^]^ Copyright 2017, The Royal Society of Chemistry. f) NIR fluorescence images of mice with different injected doses of (9, 4) SWCNTs (1.7–0 µg per mouse) under the same imaging conditions (top) and the images adjusted for optimal contrast (bottom). f) Reproduced with permission.^[^ ^55^ ^]^ Copyright 2016, Nature Publishing Group.

Light emission device based on a single SWCNT was first reported by Misewich et al. in 2003.^[^ ^287^ ^]^ The electrically driven light emission from the SWCNT‐FET has its unique characteristics, such as linearly polarized dipole radiation^[^ ^287^, ^288^ ^]^ and the wavelength‐adjustable emission.^[^ ^48^ ^]^ Due to the adjustable band structure, the emission wavelength of semiconducting SWCNTs covers a wide range of 850–2000 nm, including 850 nm, 1330 nm and even 1550 nm communication wavelengths, so they have good application prospects in the field of light‐emitting devices. Systematic study of the luminescence mechanisms of different SWCNTs is an important foundation for the preparation of high‐performance light‐emitting devices with controllable luminescence wavelength and efficiency. In 2011, Krupke's group constructed an eletroluminescenec (EL) device by employing chirality‐sorted (9, 7) SWCNTs, in which they observed an extraordinary emission performance that was assigned to excitonic E_22_ transition deriving by impact ionization, but the emission efficiency was limited.^[^ ^289^ ^]^ In 2013, Zaumseil's group obtained bright E_11_ emission in an ambipolar FET based on SWCNTs composed of various chirality species. Subsequently, they further employed near single‐chirality (6, 5), (7, 5), and (10, 5) SWCNTs to construct an electrolyte‐gated EL device and observed both exciton and redshift trion emission with tunable intensity with the applied voltages (**Figure** **17**a).^[^ ^290^ ^]^ In 2016, Liang et al. employed chirality‐sorted (8, 3) and (8, 4) SWCNTs to construct a pure trion EL device using Pd as the symmetric electrodes, in which the excitons were formed by impact excitation and rapidly charged by the plentiful surrounding holes barrier‐free injected into the channel.^[^ ^291^ ^]^

In order to effectively modulate the emission characteristics of SWCNTs, various techniques including temperature control,^[^ ^292^, ^293^ ^]^ cavity coupling,^[^ ^294^, ^295^ ^]^ covalent functionalization,^[^ ^296^, ^297^, ^298^, ^299^ ^]^ hot‐carrier injection,^[^ ^300^ ^]^ and surface interactions,^[^ ^301^ ^]^ have been developed. For example, Gaulke et al. reported that the EL FWHM of (9, 8) SWCNTs could be continuously reduced from 50 nm (30 meV) at 300 K to 5 nm (3 meV) at 4 K.^[^ ^293^ ^]^ This temperature‐dependent EL FWHM is consistent with that observed in PL measurement of various (*n, m*) SWCNTs, which can be attributed to coupling to acoustic phonons.^[^ ^292^, ^302^ ^]^ However, the requirement of such low temperature limits the practical application of EL devices. In addition to temperature, coupling SWCNTs with a micro‐resonator structure (e.g., Fabry–Pérot microcavities,^[^ ^295^, ^303^ ^]^ photonic crystals cavities,^[^ ^294^, ^304^ ^]^ plasmonic antenna,^[^ ^305^ ^]^ and so on^[^ ^20^ ^]^) is another effective way to modulate the emission characteristics of SWCNTs. Recently, Liang et al. constructed a NIR light emission device by coupling (8, 3)/(8, 4) SWCNTs with a Fabry–Pérot microcavities, and demonstrated that the EL FWHM of SWCNTs could be reduced to ≈30 nm at room temperature.^[^ ^295^ ^]^ Pyatkov et al. achieved a much narrower EL FWHM (≈1.3 nm) at room temperature by coupling SWCNTs with a photonic crystal nanobeam cavity, which is the narrowest reported so far and is very close to the spectral resolution (Figure 17b).^[^ ^294^ ^]^ In addition to FWHM, the modulation of the emission wavelength of SWCNTs into the telecom regime by coupling with a micro‐resonator structure was also reported,^[^ ^295^, ^306^, ^307^ ^]^ which is of significance for the photonic quantum information progressing and computing.

Covalent functionalization is one of most attractive approaches to modulate the emission characteristics of SWCNTs. Previously, various dopants including ozone,^[^ ^296^ ^]^ diazonium salts,^[^ ^297^, ^298^ ^]^ hydrogen peroxide,^[^ ^275^ ^]^ and phenylboronic acid,^[^ ^299^ ^]^ have been widely used to create the localized exciton states that enable to produce unique emission characteristics. An early demonstration of covalent functionalization of SWCNTs was reported by Weisman et al., in which various (*n*, *m*) SWCNTs separated by DGU method were covalently doped by ozone under the conditions of light exposure.^[^ ^296^ ^]^ Compared with the undoped SWCNTs, the emission intensity of oxygen‐doped SWCNTs was significantly enhanced, and the emission wavelength is red‐shifted. Subsequently, Wang et al. revealed that the emission enhancement factor is strongly dependent on the type of dopant and chirality of SWCNTs, in which various organic diazonium salts were used for covalent functionalization of various chirality‐separated SWCNTs.^[^ ^297^ ^]^ The maximum factor of emission enhancement (≈28‐fold) was observed for (6, 4) SWCNTs doped by 4‐nitrobenzenediazonium tetrafluoroborate.^[^ ^297^ ^]^ Recently, He et al. introduced deep‐trap states through the reaction of chirality‐separated SWCNTs with various aryl functionalization agents, and thus achieved the tunable room‐temperature single‐photon emission at telecom wavelengths (Figure 17c).^[^ ^308^ ^]^ These pioneering works have attracted great attentions, and stimulated extensive researches on the mechanisms, property, and applications of covalent functionalization of single‐chirality SWCNTs,^[^ ^275^, ^296^, ^297^, ^298^, ^299^, ^308^, ^309^, ^310^, ^311^, ^312^, ^313^, ^314^, ^315^, ^316^, ^317^, ^318^, ^319^, ^320^, ^321^, ^322^, ^323^, ^324^, ^325^, ^326^ ^]^ facilitating the development of SWCNT‐based single‐photon emission.

On the other hand, investigating the emission characteristic of functionalized SWCNTs under electrical excitation is of significance for practical applications. Most recently, Xu et al. employed functionalized (6, 5) SWCNTs to fabricate EL devices and observed the EL derived from the quantum defect formed by covalent functionalization.^[^ ^325^ ^]^ Zorn et al. systematically investigated the influence of covalent defects on charge transport within a functionalized (6, 5) SWCNT network and revealed that the defect‐derived EL emission could be tuned through the defect density.^[^ ^322^ ^]^ These findings facilitated the development of high‐performance SWCNT‐based EL devices.

Although traditional detectors based on InGaAs and HgCdTe have good sensitivity in the NIR range, a complicated and expensive cooling system is required.^[^ ^327^ ^]^ A room‐temperature‐operating NIR detector with high performance is highly desirable. SWCNTs are widely considered to be ideal materials for room‐temperature detectors due to their high carrier mobility, excellent thermal conductivity, high light absorption coefficient, and perfect compatibility with conventional semiconductor processes.^[^ ^328^, ^329^ ^]^ For this, much effort has been made to develop SWCNT‐based light detection devices in the past 20 years.^[^ ^15^, ^27^, ^111^, ^114^, ^330^, ^331^, ^332^ ^]^ Consistent with traditional photovoltaic devices, the effective separation of photon‐excited electron‐hole pairs in SWCNTs could be achieved by a built‐in field that can be constructed by chemical doping^[^ ^333^ ^]^ barrier‐free bipolar diodes,^[^ ^334^ ^]^ split gates,^[^ ^335^ ^]^ and vertical heterojunction diode geometry.^[^ ^330^, ^336^ ^]^

As early as 2010, Arnold's group employed the semiconducting‐enriched SWCNTs composed of various chirality species as the NIR active photoabsorption layer in SWCNT/C_60_ heterojunction, but the power conversion efficiency of photodetectors is very low (0.6%).^[^ ^337^ ^]^ After that, chirality‐sorted SWCNTs were successively used to obtain the target performance of photovoltaic devices due to their uniform absorption, exciton relaxation and charge generation.^[^ ^336^, ^338^, ^339^, ^340^, ^341^ ^]^ For example, Arnold et al. employed single‐chirality (7, 5) SWCNTs to construct a SWCNT/C_60_ heterojunction, and improved the power conversion efficiency.^[^ ^338^, ^339^ ^]^ Subsequent studies demonstrated that the device performances including power conversion efficiency, and external/internal quantum efficiency, could be further improved through optimization of nanotube density, film thickness, or other factors.^[^ ^339^, ^340^, ^341^ ^]^ Flavel's group employed single‐chirality (6,5) SWCNTs with optimal layer thicknesses and achieved open‐circuit voltage of 0.44 V and internal quantum efficiency of 86%.^[^ ^341^ ^]^ Most recently, they further demonstrated the potential of chirality‐sorted SWCNTs to extend the absorption of silicon solar cell, which made up the low response of silicon in the range 950–1200 nm (Figure 17d).^[^ ^340^ ^]^ Different from the photovoltaic devices of vertical heterojunction mentioned above, Liang et al. used chirality‐separated (8, 3)/(8, 4) SWCNTs to construct an asymmetric‐light‐excitation photodetector with symmetric electrical contacts.^[^ ^342^ ^]^ Unlike conventional photodetectors with symmetric contacts, the photodetectors designed in this work had unique structures that ensured that the separation of photoinduced electron‐hole pairs occurred at only one Schottky junction; hence, this device can work at zero external bias. Subsequently, they further constructed photovoltaic receivers, electrically driven transmitters, and on‐chip electronic circuits by using (8, 3)/(8, 4) SWCNTs, which were then assembled into an on‐chip 3D optoelectronic IC for optical communication between stacked functional layers.^[^ ^343^ ^]^ In SWCNT‐based photodetectors, the high‐efficient photoresponse can be obtained from single‐chirality SWCNTs at E_11_ or E_22_ transition wavelength due to wavelength‐dependent photoresponse,^[^ ^344^ ^]^ which is a key for high‐performance photodetectors. However, the limitation in the wavelength of excitation light may hinder the practical applications. To address this issue, a non‐covalently modification with different fluorescent dye molecules was developed recently by Flavel's group, the photocurrent spectroscopy clearly demonstrated that the photoresponse wavelength of dye‐sensitized single‐chirality SWCNTs was effectively extended compared with the pristine SWCNTs (Figure 17e), which facilitates the development of SWCNT‐based photodetectors.^[^ ^345^ ^]^ More descriptions on SWCNT‐based light detection devices can be found in recent review manuscripts.^[^ ^27^, ^263^ ^]^

The NIR emission wavelengths of semiconducting SWCNTs are located exactly within the biological transparency window and have strong penetration abilities in biological compounds and tissues.^[^ ^346^, ^347^, ^348^ ^]^ Separated single‐chirality SWCNTs have a promising future in bioimaging, which plays a pivotal role in the field of biomedicine and life sciences. In 2012, Diao et al. employed (12, 1) and (11, 3) SWCNTs separated by gel chromatography for biological imaging with an excitation of 808 nm and achieved 5 times higher fluorescence brightness than chirality‐mixed SWCNTs.^[^ ^349^ ^]^ Subsequently, Antaris et al. used (6, 5) SWCNTs to achieve 6 times higher fluorescence brightness due to their higher quantum yield.^[^ ^350^ ^]^ In 2016, Kataura et al. applied (9, 4) SWCNTs for the vascular imaging of mice.^[^ ^55^ ^]^ The blood vessels of the mice were clearly recognized even with an injection dose of (9, 4) SWCNTs that was 100‐fold lower than that of pristine SWCNTs (Figure 17f)^[^ ^55^ ^]^ due to their efficient light absorption and emission. Chirality‐separated SWCNTs have been demonstrated to provide drug or gene combination therapy while imaging each therapeutic component separately through specific fluorescence.^[^ ^351^ ^]^ Most recently, Mandal et al. applied biocompatible functionalized single‐chirality (6, 5) SWCNTs to in vivo fluorescence imaging and achieved higher fluorescence brightness, in which the defect‐induced emission of functionalized SWCNTs was excited by the E_11_ transition, leading to a higher signal‐to‐noise ratio under unprecedentedly low excitation intensities because both the excitation light and fluorescence are located in the NIR region.^[^ ^352^ ^]^ Because the emission wavelength of functionalized SWCNTs is redshifted compared to that of pristine SWCNTs, the influence of reabsorption on the emission intensity can also be effectively avoided,^[^ ^48^, ^353^, ^354^ ^]^ which is an advantage of functionalized single‐chirality SWCNTs. These experimental results fully prove that single‐chirality SWCNTs have broad application prospects in the field of biological imaging.

## 5 Separation of Single‐Wall Carbon Nanotube Enantiomers and Their Applications

**Figure 18:**
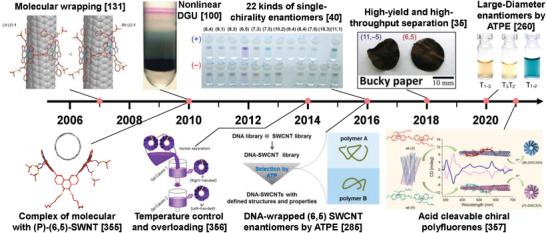
Timeline of separation of SWCNT enantiomers. Reproduced with permission from the following: Reproduced with permission.^[^ ^131^ ^]^ Copyright 2007, Nature Publishing Group; Reproduced with permission.^[^ ^100^ ^]^ Copyright 2010, Nature Publishing Group; Reproduced with permission.^[^ ^355^ ^]^ Copyright 2010, American Chemical Society; Reproduced with permission.^[^ ^356^ ^]^ Copyright 2014, American Chemical Society; Reproduced with permission.^[^ ^40^ ^]^ Copyright 2016, Nature Publishing Group; Reproduced with permission.^[^ ^285^ ^]^ Copyright 2016, American Chemical Society; Reproduced with permission.^[^ ^35^ ^]^ Copyright 2018, Elsevier Ltd.; Reproduced with permission.^[^ ^260^ ^]^ Copyright 2019, American Chemical Society; Reproduced with permission.^[^ ^357^ ^]^ Copyright 2021, American Chemical Society.

The enantiomers of single‐chirality SWCNTs not only have uniform electrical, optical, optoelectronic properties but also have unique optical activity; thus, they can be applied to chiral molecular recognition and the fabrication of chiral light‐emitting and detecting devices. The enantiomer separation of single‐chirality SWCNTs is the ultimate structure separation of SWCNTs and is more difficult than metallic/semiconducting separation and chirality separation, as it requires resolution in the handedness of the SWCNTs. To achieve enantiomer separation of SWCNTs, chiral molecules (e.g., chiral surfactants, polymers, or DNA) are generally required. If a certain chiral molecule can be adsorbed or wrapped on a pair of enantiomers of chiral SWCNTs in different ways, it is possible to achieve enantiomer separation based on the differences in their surface hydrophobicity, density, or surfactant coverage. Therefore, the modulation of the interactions of SWCNT enantiomers with chiral molecules is key to realizing the high‐purity enantiomer separation of SWCNTs. At present, the developed methods for the enantiomer separation of SWCNTs mainly include molecular wrapping, DGU, gel chromatography, and ATP (**Figure** **18**). In this section, we provide a systematic review of recent progress on the enantiomer separation of SWCNTs and their applications.

### 5.1 Molecular Wrapping‐Based Enantiomer Separation

**Figure 19:**
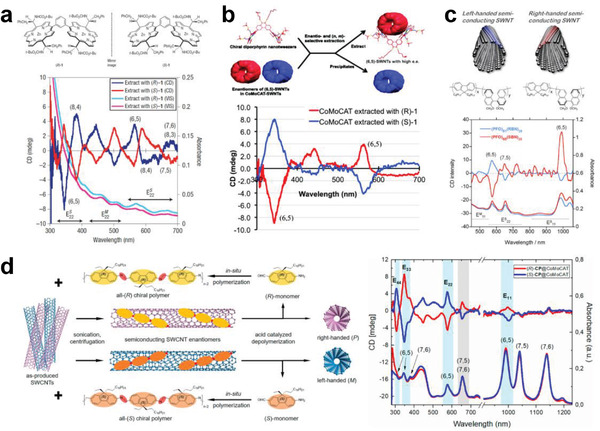
a) Molecular structure of chiral nanotweezers used for the separation of left‐handed and right‐handed SWCNT enantiomers (top), CD and optical absorption spectra of the separated SWCNT enantiomers (bottom). a) Reproduced with permission.^[^ ^131^ ^]^ Copyright 2007, Nature Publishing Group. b) Schematic of the single stereoisomer of (6, 5) SWCNT enantiomers extracted with phenanthrene‐bridged chiral diporphyrin nanotweezers (top) and the CD spectra of the separated (6, 5) SWCNT enantiomers. b) Reproduced with permission.^[^ ^355^ ^]^ Copyright 2010, American Chemical Society. c) Atomic structures of (6, 5) SWCNT enantiomers (top) and chemical structures of (PFO)*_x_* (RBN)*_y_* and (PFO)*_x_* (SBN)*_y_* (middle). Optical absorption and CD spectra of the separated (6, 5) and (7, 5) SWCNT enantiomers by (PFO)_61_(RBN)_39_ and (PFO)_65_(SBN)_35_ (bottom). c) Reproduced with permission.^[^ ^363^ ^]^ Copyright 2012, American Chemical Society. d) Schematic of the proposed separation procedure of semiconducting SWCNT enantiomers by using chiral polymers (left) and optical absorption and CD spectra of the dispersed CoMoCAT SWCNTs by chiral (R)‐ and (S)‐CP polymers. d) Reproduced with permission.^[^ ^357^ ^]^ Copyright 2021, American Chemical Society.

The molecular wrapping method has enabled the selective dispersion of the enantiomers of SWCNTs by the selective interaction of chiral molecules with them. In 2007, Komatsu's group began to develop a molecular wrapping method for the enantiomer separation of SWCNTs (**Figure** **19**a).^[^ ^131^ ^]^ They designed and synthesized a chiral zinc diporphyrin with phenyl as a bridging group (nanotweezers) for the enantiomer separation of SWCNTs (Figure 19b).^[^ ^131^, ^355^, ^358^ ^]^ More importantly, diporphyrins can be easily removed, which is very important for subsequent studies on intrinsic properties of SWCNTs and their applications. However, the separation of SWCNTs based on the chirality, diameter, or even electronic classification has not been achieved.^[^ ^131^, ^358^ ^]^ Therefore, the separated enantiomers were a mixture of SWCNTs with different structures. To enhance the selectivity, they further designed a series of diporphyrin‐based derivatives by optimizing the spacer and bridging groups.^[^ ^355^, ^359^, ^360^, ^361^, ^362^ ^]^ Among these derivatives, the nanotweezers consisting of two chiral porphyrins and phenanthrene in between showed high chiral selectivity of SWCNTs, with which the chirality and enantiomers of (6, 5) SWCNTs were separated simultaneously. They thus prepared enantiomers of single‐chirality (6, 5) SWCNTs.^[^ ^355^ ^]^

In addition to chiral diporphyrins and their derivatives, chiral FMN and PFO‐based polymers have also been demonstrated to separate the enantiomers of SWCNTs.^[^ ^129^, ^363^ ^]^ Paradimitrakopoulos and Ju et al. found that FMN containing a chiral D‐ribityl phosphate chain with a right‐handed helix can wrap around nanotubes in a helical pattern depending on the chirality and handedness of the SWCNTs.^[^ ^128^, ^129^, ^158^, ^159^, ^364^ ^]^ In addition, Nakashima et al. designed and synthesized copolymers composed of PFO and chiral binaphthol moieties for the enantiomer separation of (6, 5) and (7, 5) SWCNTs (Figure 19c).^[^ ^363^ ^]^ Most recently, Xu et al. synthesized an chiral polyfluorene that can be cleaved by acid; this polyfluorene exhibits strong recognition for SWCNT enantiomers through simple bath sonication and centrifugation (Figure 19d).^[^ ^357^ ^]^ Furthermore, chiral polyfluorene can be removed by acid treatment to obtain polymer‐free SWCNT enantiomers. However, the chirality or enantiomeric purity of SWCNTs separated by chiral FMN and polymers is still low due to their low separation resolution. To achieve high‐efficiency separation of single‐chirality SWCNT enantiomers, it is urgent to design and synthesize more chiral molecules that can better recognize the chirality and diameter of SWCNTs.

### 5.2 Density Gradient Centrifugation‐Based Enantiomer Separation

Chiral molecules also play a key role in the separation of enantiomers of single‐chirality SWCNTs by the DGU method. In 2009, Green et al. used a chiral SC surfactant to induce subtle differences in the buoyant density between a pair of two enantiomers; this was the first success in DGU‐based enantiomer separation of SWCNTs.^[^ ^365^ ^]^ However, the separable chirality species was limited to (6, 4) and (6, 5), and the chirality or enantiomeric purity was not so high that could be confirmed from their CD spectra. One year later, a significant breakthrough was reported by Ghosh et al., who used a nonlinear DGU approach to realize the enantiomer separation of 7 kinds of single‐chirality SWCNTs, in which the chiral SC surfactant was used as a cosurfactant to modulate the interactions of left‐handed and right‐handed enantiomers with cosurfactants.^[^ ^100^ ^]^ More importantly, the relative enantiomeric purities of the obtained samples, which were evaluated by the normalized CD intensity by absorbance at the E_22_ transition, were much higher than those obtained by other separation methods at that time.^[^ ^366^ ^]^ However, the quantity of single‐chirality enantiomers of SWCNTs obtained by the DGU method is limited to the microgram scale due to the complex separation and collection processes and expensive density gradient agents. These disadvantages hinder the large‐scale production and practical applications of single‐chirality enantiomers.

### 5.3 Gel Chromatography‐Based Enantiomer Separation

**Figure 20:**
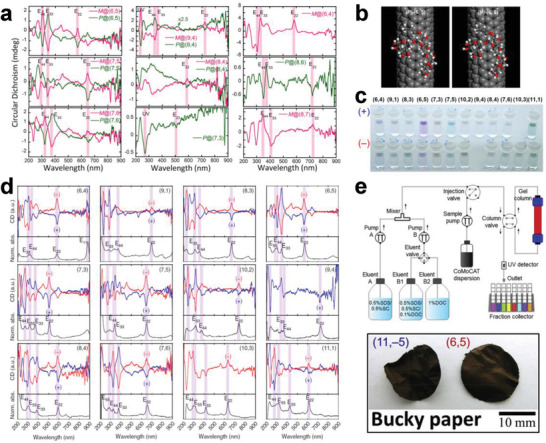
a) CD spectra of 14 kinds of separated single‐chirality enantiomers. b) Schematic of the interaction between (6, 5) SWCNT enantiomers and the chiral dextran‐based Sephacryl gel. a,b) Reproduced with permission.^[^ ^356^ ^]^ Copyright 2014, American Chemical Society. c) Photographs of 22 kinds of single‐chirality enantiomers. d) CD and optical absorption spectra of 22 kinds of separated single‐chirality enantiomers. c,d) Reproduced with permission.^[^ ^40^ ^]^ Copyright 2016, Nature Publishing Group. e) Schematic diagram of the automatic separation system of SWCNTs based on liquid chromatography (top) and photograph of the fabricated bucky papers of (11, −5) and (6, 5) SWCNT enantiomers (bottom). Reproduced with permission.^[^ ^35^ ^]^ Copyright 2018, Elsevier Ltd.

Based on the separation of single‐chirality SWCNTs, Liu et al. further applied the gel chromatography method to the separation of the enantiomers of SWCNTs. In 2014, they achieved enantiomer separation of 9 kinds of single‐chirality SWCNTs (**Figure** **20**a) in an achiral single‐surfactant SDS system by controlling the temperature and overloading.^[^ ^356^ ^]^ They revealed that the difference in the interactions between the enantiomers and the gel resulted from the chiral part of dextran‐based gels (Sephacryl S‐200), as shown in Figure 20b.^[^ ^356^ ^]^ In the absence of chiral dispersant, the interaction difference may be limited, affecting the enantiomeric selectivity. This may be a main reason for the relatively low enantiomeric purity of the separated single‐chirality SWCNTs, which was estimated from the normalized CD intensity by absorbance at the E_22_ transition. Despite this result, this work has opened up a new way to separate the enantiomers of SWCNTs.

To further improve the resolution of the enantiomers of SWCNTs by gel chromatography, Wei et al. introduced chiral SC and DOC molecules to enlarge the difference in the interaction of the enantiomers with the gel medium. In this way, simultaneous separation by chirality and by enantiomer was achieved.^[^ ^40^ ^]^ Based on this finding, 22 kinds of single‐chirality enantiomers were successfully separated, and their photographs and CD spectra are shown in Figure 20c,d, respectively.^[^ ^40^ ^]^ Compared with those separated by single surfactant‐based gel chromatography and other methods, the enantiomeric purities were greatly improved and reached the highest purity at that time. When HiPco‐SWCNTs were used as raw materials, two‐step selection procedures had to be performed, including selective adsorption by overloading and selective desorption by stepwise elution. The two‐step separation method greatly reduced the separation efficiency, and the enantiomer throughput was only on a microgram scale. To achieve the large‐scale separation of the enantiomers of single‐chirality SWCNTs, they constructed an automatic separation system based on commercial liquid chromatography equipped with a hundreds of milliliters of gel column, by which milligram‐scale enantiomer separation of (6, 5) SWCNTs was successfully achieved through one‐step stepwise elution from (6, 5)‐enriched CoMoCAT SWCNTs (Figure 20e).^[^ ^35^ ^]^ The large‐scale separation of the enantiomers of (6, 5) SWCNTs provides a new opportunity in fundamental research on SWCNT enantiomers. Most recently, enantiomer separation of functionalized SWCNTs was achieved by a one‐step stepwise elution method.^[^ ^367^ ^]^ However, due to the limitation in the type of separable enantiomers on a macroscopic scale, gel chromatography must be further improved to separate more types of enantiomers.

Similar to the chirality separation of SWCNTs, gel chromatography also has obvious advantages in the purity, species, and quantity of separable single‐chirality enantiomers compared with other separation methods, but these advantages are limited to semiconducting SWCNTs. As described above, the difficulty in the enantiomer separation of metallic SWCNTs comes from their lack of adsorption onto the gels. If suitable gel or dispersant compositions that enhance the adsorbability of metallic SWCNTs onto the gels can be discovered in upcoming studies, it will be entirely possible to achieve the simultaneous separation by chirality and enantiomers of metallic SWCNTs by precisely controlling the interactions between metallic SWCNTs and the gels under the optimized separation conditions. However, great challenges remain.

### 5.4 Aqueous Two‐Phase‐Based Enantiomer Separation

**Figure 21:**
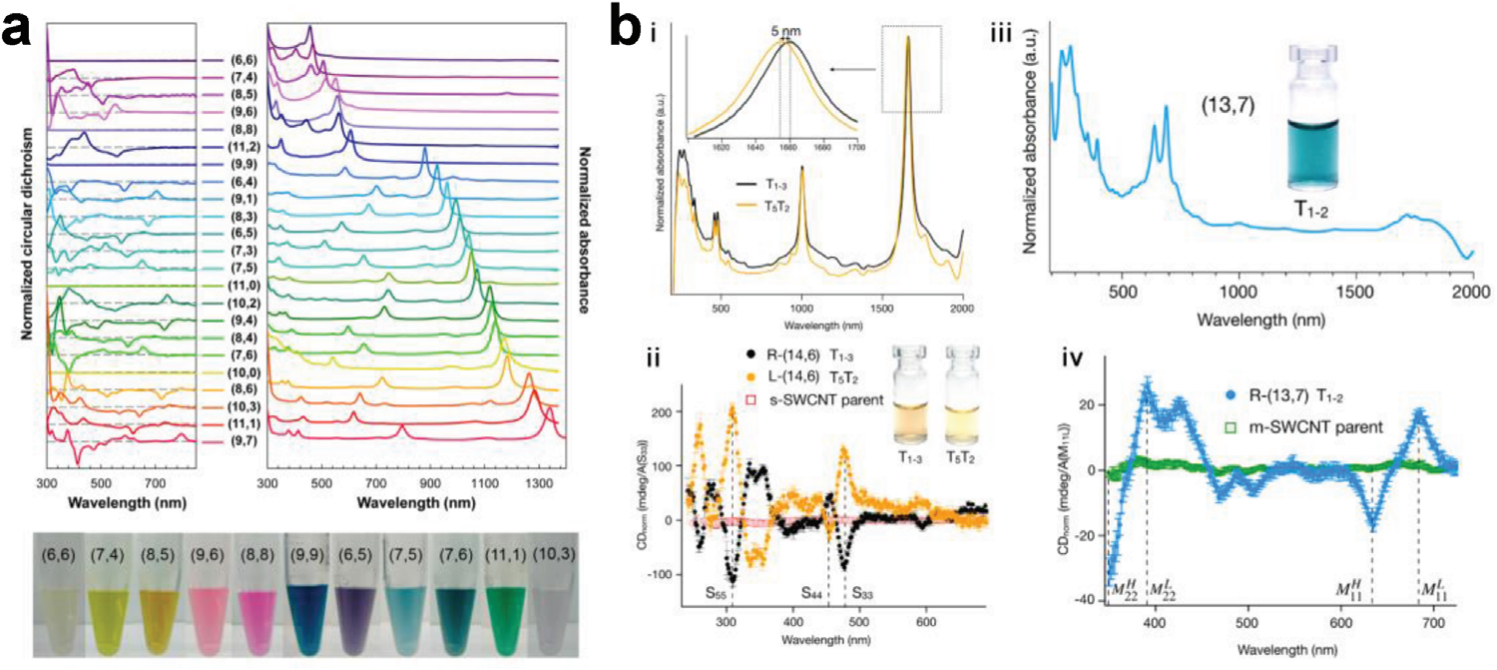
a) CD and optical absorbance spectra of separated single‐chirality SWCNTs and their enantiomers (top) and photographs of the obtained single‐chirality SWCNTs (bottom). a) Reproduced with permission.^[^ ^285^ ^]^ Copyright 2016, American Chemical Society. b) i) Optical absorbance and ii) CD spectra of separated R‐ and L‐(14, 6) SWCNT enantiomers. iii) Optical absorbance and iv) CD spectra of separated‐(13, 7) and metallic SWCNTs. b) Reproduced with permission.^[^ ^260^ ^]^ Copyright 2019, American Chemical Society.

Based on the high resolution of the two‐phase method in the metallic/semiconducting separation and chirality separation of SWCNTs, scientists have further sought to separate the enantiomers of single‐chirality species. In 2016, Ao et al. applied an ATP system to separate DNA‐wrapped SWCNTs and realized the separation of 23 kinds of single‐chirality species and their enantiomers, covering all three electronic classifications (metal, quasi‐metal, and semiconductor), as shown in **Figure** **21**a,^[^ ^285^ ^]^ and exhibiting ultrahigh sensitivity for the handedness of SWCNTs. Most of the enantiomers show very high CD intensities, indicating their high enantiomeric purities. Although only one enantiomer can be separated per pair, the types of separable single‐chirality enantiomers are the most reported thus far. It is important to point out that in this DNA system, different DNA sequences are required to separate different chirality species and their corresponding enantiomers due to the selective interaction between SWCNTs and chiral DNA molecules. To separate different types of enantiomers, it is necessary to prepare SWCNT solutions with different DNA sequences, which limits the continuity of separation procedures. To address this issue, Lyu et al. developed a new ATP system of PEG/DX with certain molecular weights (1.5 and 250 kDa for PEG and DX phases, respectively) and realized the continuous multistep separation of single chirality and its enantiomers from a single DNA‐SWCNT dispersion.^[^ ^286^ ^]^

In addition to chiral DNA, chiral surfactants were also demonstrated to be effective for enantiomer separation in the ATP system. Most recently, Li et al. used the pH‐controlled ATP method to modulate the competitive adsorption of achiral SDS, chiral DOC and SC surfactants on alkane‐filled SWCNTs and thus achieved the enantiomer separation of single‐chirality (13, 7), (14, 6), and (16, 3) SWCNTs with diameters of ≈1.4 nm (Figure 21b).^[^ ^260^ ^]^ A key point in this separation is that the recognition abilities of the chirality and enantiomer were significantly improved by using endohedral‐filled SWCNTs as the starting material for separation.^[^ ^368^ ^]^ Compared with other methods, this method exhibits unique advantages in the separation of enantiomers of single‐chirality SWCNTs with large diameters due to its ultrahigh resolution. This finding could inspire and serve as a reference for other methods of separating the enantiomers of large‐diameter single‐chirality SWCNTs. However, the filling of molecules also increases the complexity and cost of separation, which hinders their mass separation.

### 5.5 Applications of Single‐Wall Carbon Nanotube Enantiomers in Chiral Detection

The enantiomers of single‐chirality SWCNTs show highly selective interactions with chiral molecules due to their single chiral helix structure, and they can be used for the high‐precision detection and separation of chiral molecules, including proteins, DNA, and drug molecules.^[^ ^35^, ^369^, ^370^, ^371^, ^372^, ^373^, ^374^ ^]^ In addition, the unique optical activity of the enantiomers of single‐chirality SWCNTs provides a new means for the study of their optical properties. Therefore, the enantiomers of single‐chirality SWCNTs have unique application prospects in the fields of optics, biology, chemistry, and medicine.^[^ ^40^, ^355^, ^356^ ^]^ Here, we review the recent studies on the properties of SWCNT enantiomers, chiral detection, and related technological development.

**Figure 22:**
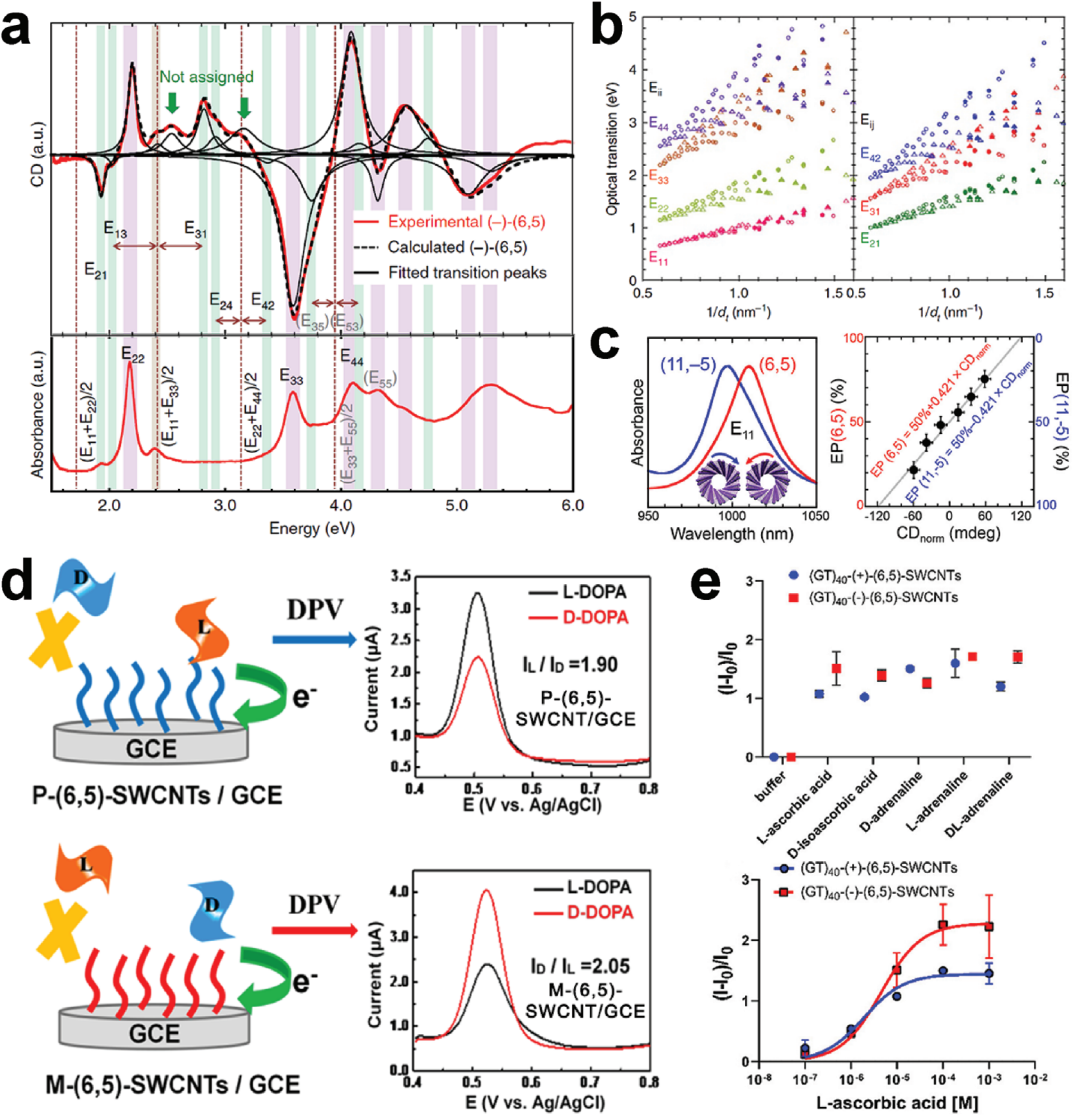
a) Assignment of E_ii_ and E_ij_ transitions (depicted as purple and green strips) in the CD (upper panel) and optical absorption spectra (lower panel) of sorted (−)‐(6, 5) SWCNTs. b) Plots of the E_ii_ and E_ij_ transition energies as a function of 1/*d*_t_. Open and solid symbols indicate the calculation and experimental results, respectively. a,b) Reproduced with permission.^[^ ^40^ ^]^ Copyright 2016, Nature Publishing Group. c) Optical absorption spectra (left) of (6, 5) and (11, −5) SWCNTs redispersed in FMN solution. Plot (right) of the enantiomeric purity as a function of the experimental CD_norm_ intensity. The gray line indicates the fitting result for the plotted enantiomeric purity values. c) Reproduced with permission.^[^ ^375^ ^]^ Copyright 2017, American Chemical Society. d) Schematic illustration (left) of the formation of chiral space on the left‐handed (denoted as M‐(6, 5)) or right‐handed (denoted as P‐(6, 5)) SWCNT‐modified GCE for the electrochemical enantiorecognition of l‐ and d‐DOPA. DPVs (right) of P‐(6, 5)‐SWCNT/GCE and M‐(6, 5)‐SWCNT/GCE in 0.25 m H_2_SO_4_ containing 25 µM l‐DOPA or d‐DOPA. d) Reproduced with permission.^[^ ^372^ ^]^ Copyright 2019, American Chemical Society. e) Plots of the (*I* − *I*_0_)/*I*_0_ value as the fluorescence response of (GT)_40_‐(+)‐(6, 5)‐SWCNTs and (GT)_40_‐(−)‐(6, 5)‐SWCNTs to stereoisomers of ascorbic acid and adrenaline (top) and dose‐response curves of l‐ascorbic acid using (GT)_40_‐(+)‐(6, 5)‐SWCNTs and (GT)_40_‐(−)‐(6, 5)‐SWCNTs (the blue and red curves indicate the fitting results) (bottom). e) Reproduced with permission.^[^ ^374^ ^]^ Copyright 2021, American Chemical Society.

In 2016, Wei et al. conducted a systematic analysis of the CD spectra of a variety of highly pure enantiomers of single‐chirality SWCNTs separated by gel chromatography (**Figure** **22**a).^[^ ^40^ ^]^ They identified the E_ii_ and E_ij_ transitions of distinct (*n, m*) species and revealed the asymmetric band structures of the valence and conduction bands by combining the prediction of density functional theory.^[^ ^40^ ^]^ Furthermore, based on the E_ij_ transition energies of different (*n*, *m*) SWCNTs, an extended empirical formula for the relationship between the optical transition energies of SWCNTs and their chiral structures was successfully established, by which the optical transition energies, including E_ij,_ of any (*n, m*) species can be estimated (Figure 22b).^[^ ^40^ ^]^

In the case of chiral‐related applications, as early as 2002, Sholl et al. used atomistic simulations to reveal the different binding energies of chiral SWCNTs with pairs of molecular enantiomers and predicted that chiral SWCNTs can be used as enantiomer‐selective adsorbents.^[^ ^369^ ^]^ Subsequently, Yoo et al. developed an SWCNT‐coated silica gel column and found that the interactions of different organic molecules with SWCNTs and their retention times in the column were highly dependent on the molecular species.^[^ ^371^ ^]^ Based on this finding, the separation of organic molecules was successfully achieved.^[^ ^371^ ^]^ Recently, functionalized SWCNTs were assembled into a thin film nanocomposite membrane and then applied for the selective permeation of a racemic mixture of tyrosine.^[^ ^373^ ^]^ The results showed that l‐tyrosine was selectively permeated, while d‐tyrosine was preferentially adsorbed on the membrane.^[^ ^373^ ^]^ After optimizing the selective properties of the SWCNT‐based membrane, including the mass loading of SWCNTs, concentration of feed, applied pressure, and reaction temperature, a higher enantiomeric excess of up to 98.86% was achieved.^[^ ^373^ ^]^ Despite this, the SWCNTs used contain both left‐handed and right‐handed enantiomers, which may limit the enantiomer selectivity of individual chiral molecules.

Based on enantiomer separation, each of the enantiomers of SWCNTs showed high selectivity for chiral molecules, including polymers,^[^ ^131^, ^355^, ^357^, ^358^, ^359^, ^360^, ^361^, ^362^, ^363^ ^]^ surfactants,^[^ ^35^, ^40^, ^100^, ^260^, ^356^, ^365^, ^375^ ^]^ and DNA.^[^ ^285^, ^286^ ^]^ The handedness‐dependent interactions of SWCNTs with chiral molecules were recently verified by optical spectroscopy, in which two SWCNT enantiomers coated with chiral FMN molecules exhibited significantly different absorption and PL wavelengths of the E_11_ optical transitions.^[^ ^375^, ^376^ ^]^ On this basis, Wei et al. established a linear relationship between the normalized E_22_ CD intensity of the enantiomers of (6, 5) SWCNTs and their absolute enantiomeric purity, which can be used as a standard to evaluate the absolute enantiomeric purity of any mixture of these two enantiomers with high accuracy (Figure 22c).^[^ ^375^ ^]^ Subsequently, similar handedness‐dependent interactions of SWCNTs with chiral DNA were also reported.^[^ ^285^, ^377^, ^378^ ^]^ Therefore, when using a single‐chirality enantiomer as a chiral medium in a column or membrane, it is possible to achieve the separation of different chiral molecules with high enantiomeric enrichment, which is an advanced application of SWCNT‐based selectors and is critical in the biochemical and medical fields.

Recently, Pu et al. developed a novel electrochemical sensor for the chiral differentiation of molecules by employing (6, 5) SWCNT enantiomers to create a chiral space on a glassy carbon electrode (Figure 22d).^[^ ^372^ ^]^ This chiral electrode demonstrated a perfect enantioselective response for 3,4‐dihydroxyphenylalanine (DOPA) enantiomers (l‐DOPA and d‐DOPA) under differential pulse voltammetry (DPV), in which the chiral electrode formed by M‐(6, 5) SWCNTs was more favorable for right‐handed d‐DOPA, while the chiral electrode formed by P‐(6, 5) SWCNTs was more favorable for left‐handed l‐DOPA (Figure 22d).^[^ ^372^ ^]^ Furthermore, this chiral electrode enabled the determination of the enantiomeric excess of DOPA based on the linear relationship between the DPV peak current and enantiomeric excess.^[^ ^372^ ^]^ This work experimentally demonstrated the potential of SWCNT enantiomers for chiral recognition and detection, providing an effective development direction in future practical applications. Most recently, single‐chirality (6, 5) SWCNT enantiomers were further used to create NIR fluorescent sensors for several important analytes, including l‐ascorbic acid, d‐isoascorbic acid, l‐adrenaline, and d‐adrenaline (Figure 22e).^[^ ^374^ ^]^ Although these studies are still in their infancy, the enantiomers of SWCNTs exhibit strongly powerful resolution in the sorting and detection of chiral molecules.

## 6 Chirality Assignment and Purity Characterization

Chirality assignment is very important in structure‐selective synthesis and separation of SWCNTs, which provides essential information for the composition analysis and subsequent purity characterization of obtained SWCNT products. The available techniques for chirality assignment can be classified into spectroscopic and microscopic approaches. In this section, we will review both spectroscopic and microscopic approaches combined with their applicability and reliability in chirality assignment and purity characterization of SWCNTs.

The current spectroscopic approaches mainly include absorption, PL, resonance Raman scattering, and Rayleigh scattering spectroscopies, in which the optical peaks derived by E_ii_ transition of SWCNTs can be observed, except for Raman spectrum that presents the diameter information (converted from the Raman shift) of SWCNTs. Based on the multiple E_ii_ transition energies or RBM shift combined with excitation wavelength, the chirality species can be assigned after referencing the plots of E_ii_ transition energies or RBM Raman shift of various chirality species (well known as “Kataura plot”) reported in previous theoretical and experimental studies.^[^ ^37^, ^38^, ^39^, ^40^, ^379^ ^]^ For example, on the basis of a pair of E_11_ emission and E_22_ excitation energies/wavelengths, we can assign the chirality species of SWCNTs contained in synthesized or separated samples. The spectroscopic methods have the advantages of simplicity, efficiency, and non‐damage, and are suitable for both liquid and solid samples, as well as, a single SWCNT and macroscopic films. Of course, each method has its own advantages and disadvantages in chirality assignment and purity characterization due to their different principles and requirements for sample preparation.^[^ ^112^ ^]^ More details will be described in the following.

**Figure 23:**
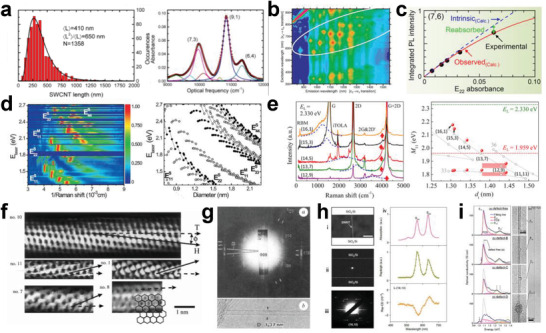
a) Length distribution of (6, 5) SWCNTs determined by nanotube diffusion analysis (left) and absorption spectra (right) after background subtraction for sample enriched in (6, 4), (7, 3), and (9, 1). a) Reproduced with permission.^[^ ^49^ ^]^ Copyright 2016, American Chemical Society. b) PL contour plot versus excitation and emission wavelengths for SWCNTs dispersed in SDS and deuterium oxide. b) Reproduced with permission.^[^ ^384^ ^]^ Copyright 2002, American Association for the Advancement of Science. c) Plot of the integrated PL intensity from experimental observation (solid dots) for (7, 6) SWCNT solution, as a function of E_22_ absorbance. Red solid and blue dashed curves indicate the fitting results for the observed and intrinsic intensities, respectively. c) Reproduced with permission.^[^ ^48^ ^]^ Copyright 2019, American Chemical Society. d) 2D contour plot (left) of RBM intensity as a function of the inverse Raman shift and laser excitation energy. The plots (right) of optical transition energies of all measured SWCNTs. The symbols corresponding to the chiralities with the same family number (2*n* + *m*) are connected to guide the eye. d) Reproduced with permission.^[^ ^379^ ^]^ Copyright 2007, The American Physical Society. e) Raman spectra (left) of metallic SWCNTs with the family number of (2*n* + *m*) = 33 at 2.330 eV excitations. The plots of estimated M_11_ transition energies (right), as a function of nanotube diameter. e) Reproduced with permission.^[^ ^395^ ^]^ Copyright 2016, American Chemical Society. f) Atomically resolved STM images of individual SWCNTs. f) Reproduced with permission.^[^ ^397^ ^]^ Copyright 1998, Macmillan Publishers Ltd. g) ED pattern (top) taken from a SWCNT with a diameter of 1.37 nm and the corresponding TEM image (bottom). g) Reproduced with permission.^[^ ^4^ ^]^ Copyright 1993, Nature Publishing Group. h) i) SEM image of a suspended nanotube across an open slit. ii) Rayleigh scattering image of the nanotube (the bright spot in the slit center). iii) TEM diffraction image of the nanotube. iv) Optical absorption (top), Rayleigh scattering (middle), and Rayleigh scattering‐CD (bottom) spectra for the nanotube. h) Reproduced with permission.^[^ ^402^ ^]^ Copyright 2021, Nature Publishing Group. i) Optical conductivities for the defect‐free region and three types of defects in a (9,2) SWCNT suspended between TEM grid. i) Reproduced with permission.^[^ ^413^ ^]^ Copyright 2018, American Chemical Society.

Absorption spectroscopy is a simple and quick way to assign the chirality of SWCNTs, in which multiple E_ii_ transitions of both metallic and semiconducting SWCNTs can be simultaneously observed by using a commercial ultraviolet–visible near–infrared spectrophotometer. The wide wavelength range is an advantage of absorption spectroscopy, which satisfies the characterization of SWCNTs with different diameters. However, for the SWCNTs containing a variety of chiralities, the spectral overlap of different (*n, m*) species may affect the precise chirality assignment and purity characterization.^[^ ^40^, ^49^, ^100^, ^255^, ^274^, ^280^, ^281^ ^]^ Such overlap becomes more serious in the optical absorption spectrum with an increase in E_ii_ transition order (ii value). This is the reason why the peak decomposition is usually carried out for E_11_ transition, but not for higher transitions. In this case, to identify the chirality distribution precisely, PL or Raman spectroscopy are usually used as auxiliary tools for structure characterization. Notably, when absorption spectroscopy is used to evaluate the chirality purity of SWCNTs, another two issues should be considered: one is background absorption,^[^ ^380^, ^381^ ^]^ and the other one is chirality‐dependent absorption cross section (**Figure** **23**a),^[^ ^44^, ^45^, ^46^, ^49^ ^]^ which may affect the chirality purity evaluation. The absorption background usually derives from impurities such as amorphous carbon and catalyst particles in the sample, which can be purified and removed by centrifugation or separation.^[^ ^145^ ^]^ The remained low background could be further removed by conventional spectral background subtraction, or by using an empirical equation corresponding to the absorption background developed by Weisman et al.^[^ ^381^ ^]^ After removing the absorption background, the purity of each (*n*, *m*) species is generally calculated as an integrated intensity ratio of the E_11_ absorption peak of the target chirality to the sum of all other E_11_ peaks. In this case, however, the estimated value only reflects the optical purity, which should be further corrected by the absorption cross section of each chirality species which is different for distinct (*n, m*) species.^[^ ^40^ ^]^ The absorption spectroscopy has been sufficiently proved to be reliable in the evaluation of the concentration, structure identification and chirality purity of SWCNTs.^[^ ^265^, ^382^, ^383^ ^]^

PL spectroscopy is another effective way to assign the chirality for semiconducting SWCNTs, but not for metallic SWCNTs due to the nonradiative exciton recombination induced by their continuous density of states (DOS) near the Fermi level. In the classic 2D spectral mappings on E_22_ excitation‐E_11_ emission that was first developed by Weisman et al.,^[^ ^384^ ^]^ both E_11_ and E_22_ transition wavelengths of various chirality species contained in HiPco SWCNTs can be easily identified, due to the higher spectral resolution than absorption spectroscopy (Figure 23b). Furthermore, the measured PL intensity of each species is comparable after total correction for the sensitivity of the InGaAs detector, transmittance of the monochromator, and efficiency of the other optical components in PL measurement equipment, by calibration function obtained using a standard lamp.^[^ ^48^ ^]^ Because of these advantages, the PL intensity is also often used to evaluate the chirality purity of as‐grown or chirality‐sorted SWCNTs.^[^ ^70^, ^260^, ^385^, ^386^, ^387^, ^388^ ^]^ For the purity estimated by the PL spectroscopy, however, several issues, such as, chirality‐dependent PL quantum yield,^[^ ^43^, ^47^, ^48^ ^]^ reabsorption effects,^[^ ^48^ ^]^ PL susceptibility to environment,^[^ ^285^, ^389^ ^]^ and PL from oxidized states,^[^ ^275^, ^296^, ^312^ ^]^ have to be considered, which could modify the PL spectra (e.g., shape and intensity) and affect the purity evaluation. For example, the experimentally measured PL intensities of different chirality species cannot directly reflect their true abundance, because the PL quantum yields of different (*n, m*) species may varies by more than an order of magnitude.^[^ ^43^ ^]^ Wei et al. experimentally observed that the PL intensity of SWCNTs could be greatly modified by the reabsorption effect, resulting that the observed PL intensity was inconsistent with the intrinsic PL intensity (Figure 23c).^[^ ^48^ ^]^ Therefore, a correction by the chirality‐dependent quantum yield and the reabsorption effect has to be performed for the quantitative evaluation of the chirality distribution or purity of SWCNTs based on PL spectroscopy.

Raman spectroscopy is one of the most promising ways to characterize the structures and properties of SWCNTs, which provides various useful information, such as electronic types, diameter or chirality distributions, defect of functionalization content, and so on. Generally, the RBM shift combined with the resonance excitation wavelength allows us to assign the chirality species in as‐grown or chirality‐separated samples. Similar to PL spectroscopy, the higher spectral resolution than absorption spectroscopy is an important advantage for resonance Raman spectroscopy. Conversely, in the Raman measurements coupled with one laser with specific excitation wavelength, only limited types of chirality species can be excited and the resonance efficiency of each excited species is different, which is unfavorable for the evaluation of chirality distribution and purity of the measured samples by the comparison of their Raman intensities. In contrast, the 2D contour maps of RBM intensity as a function of the Raman shift and laser excitation energy can address this issue, but the wavelength‐tunable lasers and complex calibration of Raman intensity in the visible and infrared regions are indispensable. Previously, Jorio et al. demonstrated a comprehensive detection for over 200 different chirality species with a broad diameter distribution of 0.7–2.3 nm (Figure 23d).^[^ ^379^ ^]^ Through the 2D Raman spectral maps, the chirality purity can be evaluated by the integrated RBM intensities of different species after considering the chirality‐dependent Raman cross section.^[^ ^253^, ^269^ ^]^ For SWCNT solutions, a quantitative correction of the reabsorption effect is also very important for the evaluation of the chirality distribution measured by the Raman spectroscopy.^[^ ^354^ ^]^ Despite this, the Raman spectral features may also be affected by many parameters, such as bundles, dielectric environment, temperature, and pH, which should be additionally considered during chirality assignment and purity characterization.^[^ ^390^, ^391^, ^392^ ^]^ In addition to RBM, the electronic Raman scattering (ERS) is another interesting feature in Raman spectra, which is a resonance enhancement process and occurs when the scattered photon energy is close to the M_ii_ transition energy of metallic SWCNTs.^[^ ^393^ ^]^ Recently, Yang et al. systematically investigated the ERS features of various metallic chirality species, and then estimated the corresponding transition energy of M_11_ and M_22_ from ERS, achieving the accurate chirality assignment for metallic SWCNTs (Figure 23e).^[^ ^394^, ^395^ ^]^ This newly developed approach was verified to have good suitability in complex environments.^[^ ^396^ ^]^

The current microscopic approaches mainly include scanning tunneling microscopy (STM) and high‐resolution transmission electron microscopy (HRTEM), both of which provide the atomically resolved images including the information of nanotube diameter *d*_t_ and chiral angle *θ* for chirality assignment. In 1998, Dekker's group first performed the STM measurement for individual SWCNTs suspended on the flat Au surface, and obtained an accuracy of ≈0.05 nm for *d*_t_ and ≈1° for *θ*, achieving an unambiguous chirality assignment (Figure 23f).^[^ ^397^ ^]^ Similar results were also reported by Lieber's group at the same time.^[^ ^398^ ^]^ When the individual SWCNTs were suspended on a graphite surface, Zhang et al. employed STM to further determine the handedness (enantiomer) and *θ*, according to the helicity‐dependent SWCNT alignment on graphite.^[^ ^399^, ^400^ ^]^ In most recent researches, STM was also employed to identify the chirality or handedness of SWCNTs.^[^ ^401^, ^402^ ^]^ In addition to atomically resolved observation, the STM technique can be used to further reveal the electronic structures (DOS) of SWCNTs by measuring the *I*–*V* curve.^[^ ^397^, ^398^, ^403^ ^]^ Although there is an error of 50–100 mV,^[^ ^397^ ^]^ the STM technique has been demonstrated to be an effective approach for the experimental determination of the band structures of SWCNTs. Compared with 2D layered nanomaterials, such as graphene^[^ ^404^ ^]^ and borophene,^[^ ^405^ ^]^ the STM measurements is more complex for 1D SWCNTs because the magnitude of tunneling current is strongly dependent on the distance between STM probe and carbon atoms, which is the reason for the clearer stripe for middle carbon atoms than for carbon atoms on both sides.

Similar to STM, HRTEM can also determine *d*_t_ and *θ* of SWCNTs, and thus achieve their chirality assignment.^[^ ^406^ ^]^ In an aberration‐corrected HRTEM demonstration, the moiré pattern formed by the rolled‐up graphene layer can be used to estimate the nanotube diameter, while the Fourier transform derived from the HRTEM image can be used to estimate the chiral angle based on two symmetric hexagons corresponding to the front and back walls of nanotube with a mismatch angle (2*θ*).^[^ ^406^ ^]^ However, both *d*_t_ and *θ* estimated by HRTEM contain a non‐negligible error (e.g., ≈3% for *d*_t_ and ≈3° for *θ*).^[^ ^406^ ^]^ The electron diffraction (ED) is another effective way to identify the chirality of SWCNTs, which was first employed to characterize SWCNTs by Iijima and Ichihashi when they discovered SWCNTs (Figure 23g).^[^ ^4^ ^]^ Later, a serious of ED‐based methods was developed to determine the chirality of SWCNTs by using the layer‐line intensity and spacing in ED pattern, which have been reviewed in previous paper.^[^ ^407^, ^408^, ^409^, ^410^, ^411^ ^]^ However, the accuracy of *d*_t_ and *θ* determined by HRTEM and ED measurements is usually affected by the inclination of target nanotubes to the vertical electron beam, and thus a careful correction of the inclination is required. In a word, the accurate chirality assignment of SWCNTs requires ultrahigh spatial resolution of equipment, as well as, high sample preparation and measurement skills. Different from spectroscopic approaches, microscopic methods for the purity characterization of SWCNTs requires a large number of measurements and subsequent statistical analysis, which is a time‐consuming and boring process. Because the microscopic characterization is to measure the local atomic structure on a SWCNT, the characterization for even the same SWCNT is uncertain (e.g., chirality alteration along its axis^[^ ^412^ ^]^). Therefore, the microscopic approaches are not widely used for the purity evaluation of SWCNTs, but employed as the auxiliary support of spectroscopic characterization. The microscopic approaches are often used to characterize atomic structures such as defects because of their atomic scale resolution.^[^ ^406^, ^413^ ^]^

In fact, to improve the characterization accuracy, some new characterization techniques based on the combination of spectroscopy and microscopy, have been developed.^[^ ^402^, ^413^, ^414^, ^415^, ^416^, ^417^ ^]^ For example, Kappes et al. combined AFM with the PL microscopy to investigate the topographical and spectroscopic information of individual SWCNTs on sapphire.^[^ ^414^ ^]^ Similar technique was used for the chirality assignment and purity evaluation of SWCNTs by Weisman et al.,^[^ ^415^ ^]^ but one by one measurement is required. Most recently, Liu et al. developed a Rayleigh scattering CD spectroscopy that has a significantly enhanced signal compared with conventional absorption CD spectroscopy.^[^ ^402^ ^]^ This approach has been proven to be effective for the complete structural characterization of individual SWCNTs including chirality and handedness (Figure 23h).^[^ ^402^ ^]^ In addition, the combination of electron energy‐loss spectroscopy and TEM by Suenaga et al. exhibit excellent ability for the investigation of the optical conductivities of defect‐modulated SWCNTs (Figure 23i).^[^ ^413^ ^]^ Other approaches, such as FET,^[^ ^57^, ^418^ ^]^ scanning probe microscopy,^[^ ^419^ ^]^ and scanning electron microscopy,^[^ ^420^, ^421^ ^]^ were also developed to identify the conductivity (metallic and semiconducting) of SWCNTs, but it is still unavailable for chirality assignment. Despite this, the continuous development of various characterization techniques lays an important foundation for the synthesis, purification, and applications of SWCNT materials.

## 7 Removal of Dispersant Molecules

As mentioned above, dispersant plays a key role in the dispersion and separation of SWCNTs. However, dispersant is also a double‐edged sword, which has a negative effect on the application of the separated SWCNTs in electronic and optoelectronic devices. The dispersant molecules such as polymer, surfactant and DNA wrapping around the separated SWCNTs would increase nanotube‐electrode contact resistance and nanotube‐nanotube junction resistance due to their low conductivity and scattering effect and thus greatly degrade device performances. This is one of the main reasons why transparent conductive films prepared by solution‐processed SWCNTs show higher resistance than those by growth except for shortening effect mentioned in Section 2.^[^ ^18^, ^30^, ^32^, ^176^, ^177^, ^422^, ^423^, ^424^, ^425^, ^426^ ^]^

To remove the dispersant molecules, various techniques have been developed, including rinsing,^[^ ^427^, ^428^ ^]^ filtration,^[^ ^429^, ^430^ ^]^ oxidation,^[^ ^431^, ^432^ ^]^ annealing,^[^ ^240^, ^433^, ^434^ ^]^ and so on. The rinsing and filtration approaches can remove most of the free dispersant molecules, but difficultly remove those tightly wrapped around nanotubes through *π*–*π* interactions, as demonstrated by TEM.^[^ ^432^ ^]^ The oxidation, which selectively etches surfactants and polymers through acid or oxygen, has proved to be an effective method to remove dispersants.^[^ ^435^ ^]^ For example, the conductivity of the transparent conductive SWCNT film was significantly improved after the removal of dispersants by oxidation.^[^ ^436^ ^]^ However, with this technique, it is difficult to strike a balance between the complete removal of dispersant molecules and the prevention of oxidative damage to carbon lattice. The structure of SWCNTs is easily damaged during intense oxidation, resulting in undesired defects. The annealing in vacuum or inert gas is another attractive method to remove dispersant molecules because of their lower decomposition temperature than that of SWCNTs.^[^ ^240^, ^436^, ^437^, ^438^, ^439^, ^440^ ^]^ Most recently, a rapid annealing and cooling approach (rise from room temperature to 600 °C within 5 s) was recently developed,^[^ ^441^ ^]^ with which the electrical performances of semiconducting SWCNT films were significantly improved compared with those films rinsed with an organic solvent. Specifically, the contact resistance between nanotube and electrode was reduced by 700%, and the on‐current was increased by 600%. The rapid annealing method can remove most of polymers wrapping around SWCNTs due to the significant difference in the thermal expansion coefficient between polymers and SWCNTs. Moreover, the damage of SWCNT structures can be effectively avoided. In order to effectively remove dispersants, several research groups have recently designed and synthesized biodegradable polymer molecules for the separation of SWCNTs.^[^ ^192^, ^193^, ^194^ ^]^ However, some decomposition or degradation products, such as aromatic backbone of conjugated polymers, are still difficult to remove.^[^ ^441^ ^]^ Similar problem is also unavoidable for surfactants and other dispersant molecules, as demonstrated by thermogravimetric analysis.^[^ ^436^, ^442^, ^443^, ^444^, ^445^ ^]^ Therefore, the complete removal of dispersant molecules still remains a challenge.

## 8 Summary and Outlook

Over the past twenty years, a library of dispersants that is compatible with the dispersion and separation of SWCNTs was developed. Using these dispersants, great progress in separation techniques has been achieved, allowing us to separate semiconducting/metallic, chirality, and even enantiomers of SWCNTs. Among them, polymer wrapping, gel chromatography, and ATP are highly capable of separating well‐defined SWCNTs, but they have different advantages. The polymer wrapping method exhibits higher selectivity in the separation of metallic and semiconducting SWCNTs and lower separation resolution for chirality and enantiomers. In contrast, the gel chromatography and ATP methods show high resolution in the separation of single‐chirality species and even their enantiomers. In particular, gel chromatography is capable of separating highly pure single‐chirality SWCNTs on the milligram scale, indicating that it has great development prospects in industrial preparation. These achievements have greatly promoted the application of SWCNTs and the development of the techniques of purity characterization and dispersant removal. The future development of separation techniques can grow from the advantages of various current techniques to further improve the resolution of SWCNT structures and separation efficiency, finally realizing the enantiomer separation of SWCNTs on the industrial scale to satisfy the requirements of practical applications in electronics, optics, optoelectronics and bioimaging.

Compared with semiconducting/metallic and chirality separation, progress in property studies and application exploration of SWCNT enantiomers is lagging behind. There are two main reasons: One is the ultrahigh technical requirements in material preparation as described above, and the other is the unknown absolute enantiomeric purity for individual chirality species. Previously, scientists used the normalized CD intensity by absorbance at the E_22_ transition to evaluate the relative enantiomeric purity of separated single‐chirality SWCNT enantiomers, where a higher CD intensity indicated a higher relative enantiomeric purity, but the absolute enantiomeric purity remained unclear.^[^ ^40^, ^100^, ^285^, ^355^, ^356^ ^]^ Although a few methods have been developed recently to evaluate the absolute enantiomeric purity of separated SWCNT enantiomers,^[^ ^285^, ^375^, ^376^ ^]^ the variety of applicable chiralities and the evaluation accuracy are very limited. Standardization of the quantitative evaluation of the absolute enantiomeric purity of SWCNTs is still urgently desired. Despite these limitations, with the development of diverse recognition and characterization techniques of SWCNT enantiomers, such as Raman optical activity spectroscopy and Rayleigh scattering CD spectroscopy,^[^ ^402^, ^446^ ^]^ more unforeseeable properties and applications of SWCNT enantiomers will emerge in the near future.

In addition to the structure separation and applications of SWCNTs, there are three main challenges: 1) The complete removal of dispersant molecules (e.g., surfactants, polymers, or DNA) from the nanotube surface; 2) their arrangement on the target substrate; and 3) property modulation and functional design toward specific advanced applications, which are critical intermediate links that connect the structure separation and applications of SWCNTs and are of significance for realizing the practical applications of SWCNTs. From our perspectives, a real opportunity to use structure‐controlled SWCNTs to realize more advanced applications, especially in next‐generation optoelectronic technologies, has undoubtedly arrived.

## Conflict of Interest

The authors declare no conflict of interest.
